# Projected Savings From Reducing Low-Value Services in Medicare

**DOI:** 10.1001/jamahealthforum.2025.3050

**Published:** 2025-08-01

**Authors:** David D. Kim, A. Mark Fendrick

**Affiliations:** 1 Department of Medicine, Biological Sciences Division, University of Chicago, Chicago, Illinois; 2 Department of Public Health Sciences, Biological Sciences Division, University of Chicago, Chicago, Illinois; 3 Department of Internal Medicine, University of Michigan Medical School, Ann Arbor; 4 Department of Health Management and Policy, University of Michigan School of Public Health, Ann Arbor

## Abstract

This cross-sectional study estimates the utilization and potential savings achievable by reducing 47 low-value services used by Medicare beneficiaries.

## Introduction

Identifying strategies to effectively reduce Medicare spending has been challenging. Medicare’s innovative payment and delivery models have yielded limited savings due to design complexities.^1^ Moreover, some approaches, such as those proposed by the Congressional Budget Office to substantially reduce Medicare expenditures,^2^ may carry risks of unintended consequences for patient health. One promising approach to achieve savings without compromising patient health is reducing spending on medical services that offer little to no clinical benefit and potentially cause harm (ie, low-value services).^3^ We estimated the utilization and potential savings achievable by reducing 47 low-value services used by Medicare beneficiaries.

## Methods

We conducted a retrospective cohort study using a 5% random sample of 2018-2020 Medicare fee-for-service claims from beneficiaries 65 years and older with continuous 12-month enrollment. Based on our updated review of claims-based algorithms from literature, we evaluated 47 low-value services, including screenings, diagnostic tests, imaging, and procedures (eMethods in Supplement 1). After identifying eligible beneficiaries indicated for each service (denominator), we measured the number who received the low-value service (numerator). We applied stringent eligibility criteria (ie, more specific low-value care definitions) to minimize misclassification of clinically necessary services. For example, prostate-specific antigen (PSA) screening for men 70 years and older was not considered low value for those with previously elevated PSA levels or a personal/family history of prostate cancer.

We also quantified potential annual savings associated with low-value services based on Medicare payments and beneficiary out-of-pocket payments. To estimate nationwide impact, we extrapolated findings to the entire Medicare population (65.7 million). Additionally, we analyzed spending on 6 commonly used low-value preventive services (chronic obstructive pulmonary disease screening, bacteriuria screening, PSA tests for prostate cancer screening, screening for asymptomatic carotid artery stenosis, and electrocardiogram for cardiac screening) that received a grade D recommendation from the US Preventive Services Task Force (USPSTF).

This study followed the STROBE reporting guidelines for cross-sectional studies and was approved by the Tufts Medical Center/Tufts University institutional review board. Data were analyzed from January to December 2023.

## Results

**Figure 1.**
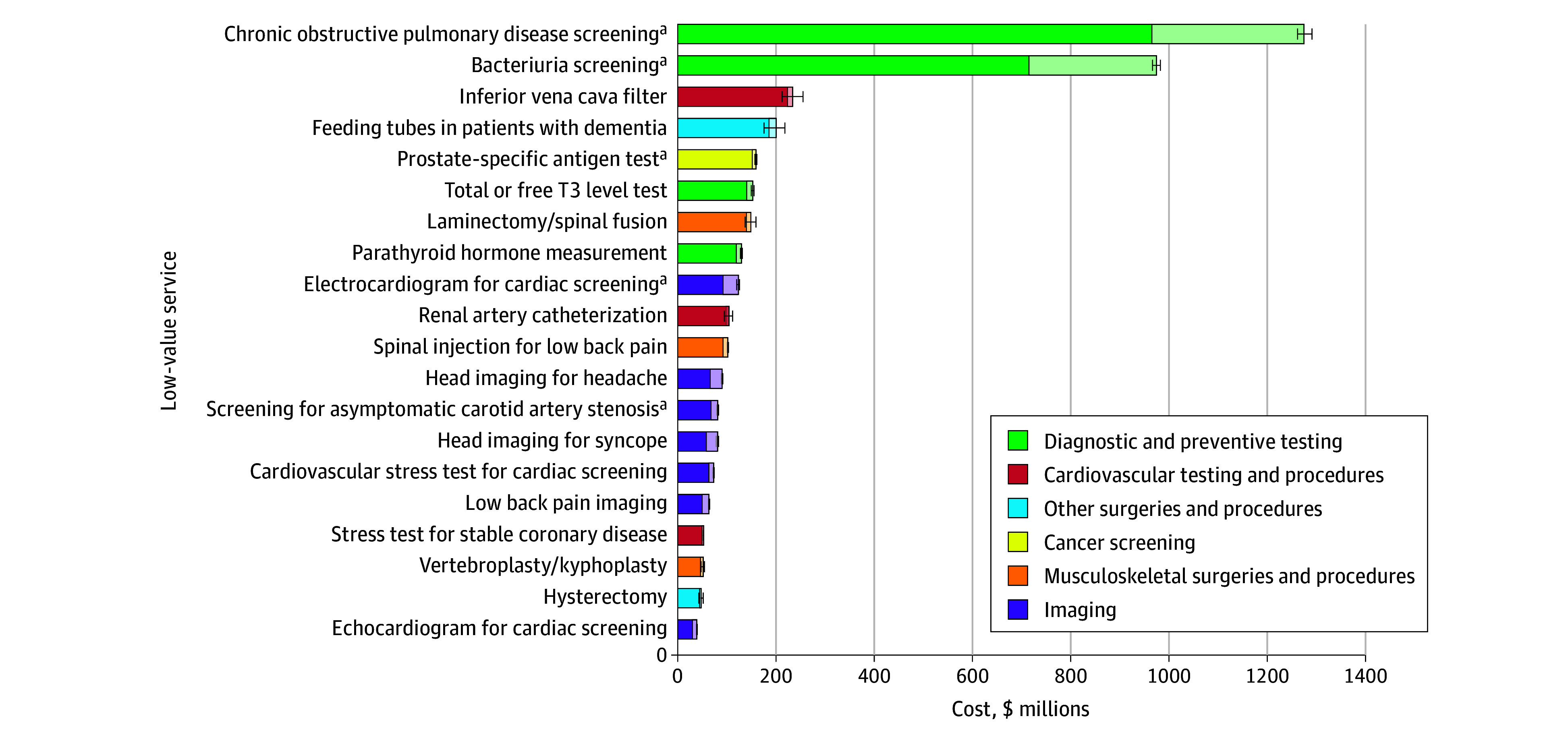
Potential Annual Savings Associated With Top 20 Low-Value Services Among Medicare Beneficiaries The analysis was based on annualized spending associated with the top 20 low-value services from the 2018-2020 Medicare fee-for-service claims data. Since the top 20 services accounted for 95% of the total annual spending ($4.2 billion of $4.4 billion), the figure presents the results among the top 20 services. Lighter areas of the bars represent out-of-pocket costs, and error bars represent 95% CIs. T3 indicates triiodothyronine. ^a^Services with US Preventive Services Task Force grade D recommendations.

**Figure 2.**
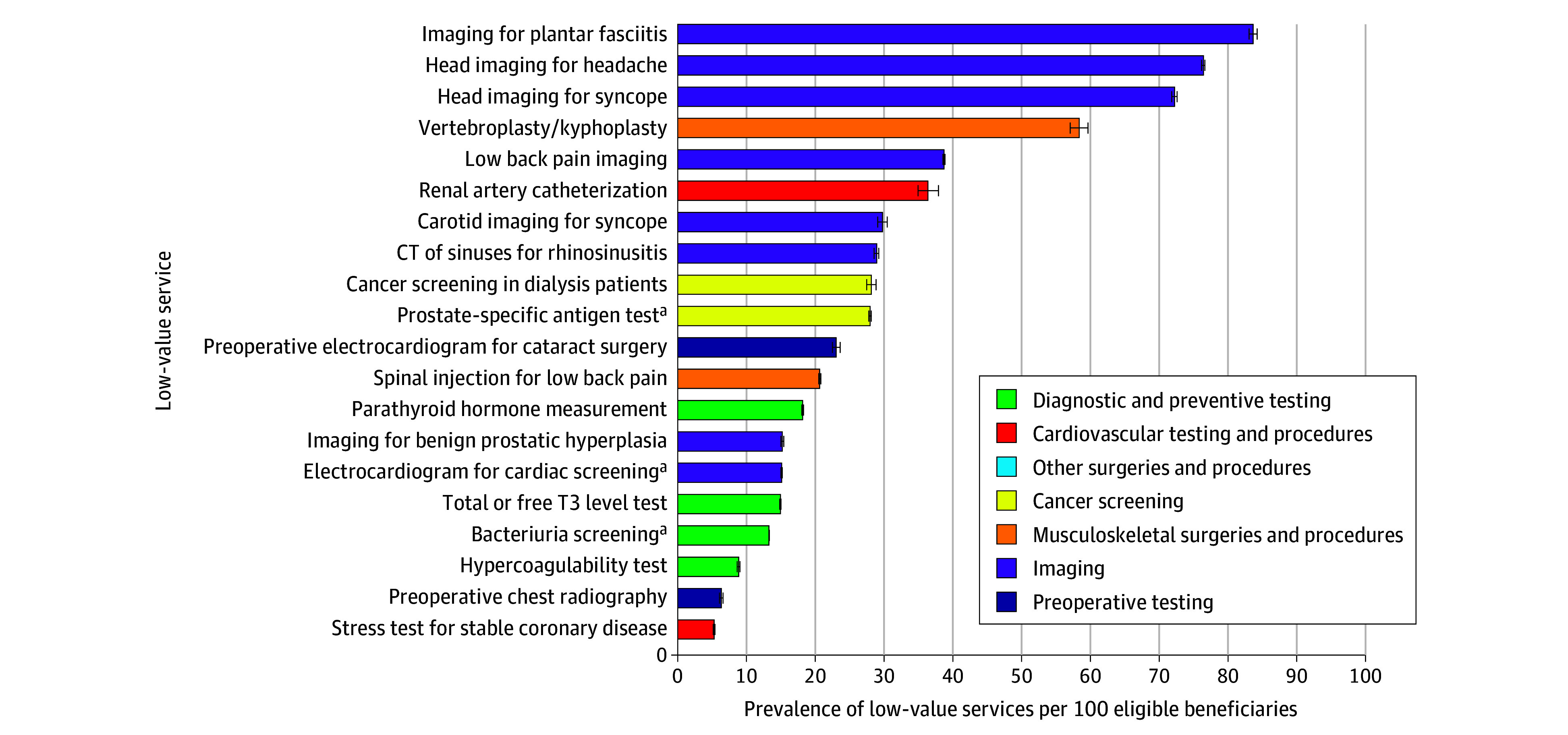
Top 20 Most Prevalent Low-Value Services Among Medicare Beneficiaries, 2018-2020 The analysis was based on the annual prevalence of the top 20 low-value services from the 2018-2020 Medicare fee-for-service claims data. Since the top 20 services accounted for 94% of the total prevalence (622 of 663 per 100), the figure presents the results among the top 20 services. Error bars represent 95% CIs. CT indicates computed tomography; T3, triiodothyronine. ^a^Services with US Preventive Services Task Force grade D recommendations.

Based on claims of 3.7 million beneficiaries, Medicare annually spent $3.6 billion across 2.6 million cases of the 47 low-value services between 2018 and 2020, with an additional $800 million annually paid from out-of-pocket payments. The top 20 services accounted for 95% of total annual spending ($4.2 billion of $4.4 billion; Figure 1). Five USPSTF grade D services among these were responsible for 59% ($2.6 billion), with chronic obstructive pulmonary disease and bacteriuria screening being the most costly. Four of the top 5 most frequently provided services were low-value imaging for conditions such as plantar fasciitis, headache, syncope, and low back pain (Figure 2).

## Discussion

Findings of this cross-sectional study highlight that reforms aimed at reducing spending for low-value services are feasible to achieve billions in savings without compromising patient health. For example, eliminating Medicare payments for 5 USPSTF grade D services could save approximately $2.6 billion annually, achievable through enforcing section 4105 of the Affordable Care Act, which grants the secretary of the US Department of Health and Human Services the authority to provide no payment for a preventive service that has not received a USPSTF grade of A, B, C, or I.^4^

Of note, the estimated savings are considered conservative, since additional costs from subsequent care cascades following low-value interventions are not included. A previous study found that every dollar spent on low-value PSA screening resulted in an additional $6 spent on related care cascades among Medicare Advantage enrollees.^5^ Implementing careful prior authorization could further deter unnecessary diagnostic testing or procedures (eg, preoperative evaluations in low-risk surgeries), potentially increasing savings and preventing harm.^6^

All of the estimated savings may not be fully realized due to gaming claims-based rules (eg, head imaging for syncope gets coded as head imaging for a fall or trauma) or substitution of other services that might be pursued instead of low-value services. Nevertheless, reducing payments for low-value services could lead to substantial savings to create headroom to pay for high-value services while preserving the health of the Medicare population.

## Notes

Supplement 1.
eMethods

eReferences

Supplement 2.
Data Sharing Statement
