# Supplementary material for: Projected Savings From Reducing Low-Value Services in Medicare
Source: JAMA Health Forum. 2025 Aug 1;6(8):e253050. doi: 10.1001/jamahealthforum.2025.3050 (PMC12317346; doi:10.1001/jamahealthforum.2025.3050)
Supplement: Supplement 1. — eMethods eReferences [file jamahealthforum-e253050-s001.pdf]

Supplemental Online Content

Kim DD, Fendrick AM. Projected savings from reducing low-value services in Medicare. *JAMA Health Forum*. 2025;6(8):e253050. doi:10.1001/jamahealthforum.2025.3050

eMethods

eReferences

This supplemental material has been provided by the authors to give readers additional information about their work.

© 2025 Kim DD et al. *JAMA Health Forum*.

eMethods.

Low-Value Care Claims-Based Algorithms

Low-Value Service Definitions

The numerator represents the service; the denominator represents the patient population for which the service would be low-value. Both have inclusion and exclusion criteria of codes used to identify instances in medical claims data.

Sources

We used multiple sources to define low-value services. Clinical sources are recommendations from the United States Preventative Services Task Force (USPSTF) and Choosing Wisely (CW)* and were identified via low-value study sources at the time of review (August 2022) Low-value study sources are other peer-reviewed publications that examine low-value care. The tables below include the sources that informed each definition. We considered the recency of publication, comprehensiveness of medical codes presented, alignment across sources, study purpose/context, and other considerations when creating definitions for this analysis. All sources are listed in order of publication year.

Sensitivity Analyses

In sensitivity analyses, we expanded the definition of patients for whom the service would be low-value. We either added inclusion criteria or removed exclusion criteria from the denominator. Changes made for sensitivity analyses are in red text. Not all low-value service definitions have a definition for sensitivity analyses.

*The US Choosing Wisely campaign was discontinued in 2023, and recommendations from that source are no longer publicly available via Choosing Wisely.

© 2025 Kim DD et al. *JAMA Health Forum*.

1. Cervical cancer screening

Recommendation	Clinical Source(s)	Low-Value Study Source(s)
Do not screen women age 65 or older for cervical cancer if they have had adequate prior screening* and are not otherwise at high risk for cervical cancer.<br>*We assume all patients had adequate prior screening if over age 65	CW Canadian Association of Pathologists (2021), CW Society of Obsetetricians and Gynaecologists of Canada (2021), CW College of Family Physicians of Canada (2020), USPSTF Melnikow (2018), CW American Academy of Family Physicians (2013)	Ganguli (2021)[1], Park (2021)[2], Sanghavi (2021)[3], Bouck (2018)[4], Schwartz (2018)[5], Carter (2017)[6], McAlister (2017)[7], Pendrith (2017)[8], Schpero (2017)[9], Colla (2015)[10]

Definition – Base Case		Codes	Include	Exclude
**Numerator**	Patients that received cervical cancer screening	ICD-10		
		BETOS		
		CPT/HCPCS	G0123, G0124, G0141, G0143, G0144, G0145, G0147, G0148, P3000, P3001, Q0091, 88141, 88142, 88143, 88147, 88148, 88150, 88152, 88153, 88154, 88155, 88164, 88165, 88166, 88167, 88174, 88175	
		MS-DRGs/Drug		
		Time	On claim	
		Age		
		Gender		
		Other		
**Denominator**	Women age 65 or older **and** not at high risk for cervical cancer<br>**High risk indicators:** history of cancer or dysplasia, diagnoses of other female genital cancers, abnormal Pap findings, HPV, diethylstilbestrol exposure, HIV/AIDS	ICD-10		B20-B24, C51, C52, C53, C57, D06, D07.0, D07.1, D07.2, D07.3, D26.0, N87, P04.8, R87, Z77.9, Z85.40-Z85.44, Z87.41
		BETOS		
		CPT/HCPCS		
		MS-DRGs/Drug		
		Time	On claim	All past data
		Age	65+	
		Gender	Female	
		Other		

© 2025 Kim DD et al. *JAMA Health Forum*.

Definition – Sensitivity Analysis		Codes	Include	Exclude
**Numerator**	Patients that received cervical cancer screening	ICD-10		
		BETOS		
		CPT/HCPCS	G0123, G0124, G0141, G0143, G0144, G0145, G0147, G0148, P3000, P3001, Q0091, 88141, 88142, 88143, 88147, 88148, 88150, 88152, 88153, 88154, 88155, 88164, 88165, 88166, 88167, 88174, 88175	
		MS-DRGs/Drug		
		Time	On claim	
		Age		
		Gender		
		Other		
**Denominator**	Women age 65 or older **and** are not at high risk for cervical cancer<br>**High risk indicators:** history of cancer or dysplasia, diagnoses of other female genital cancers, abnormal Pap findings, HPV	ICD-10		C51, C52, C53, C57, D06, D07.0, D07.1, D07.2, D07.3, D26.0, N87, R87, Z85.40-Z85.44, Z87.41
		BETOS		
		CPT/HCPCS		
		MS-DRGs/Drug		
		Time	On claim	All past data
		Age	65+	
		Gender	Female	
		Other		

© 2025 Kim DD et al. *JAMA Health Forum*.

2. Colorectal cancer screening

Recommendation	Clinical Source(s)	Low-Value Study Source(s)
Do not perform colorectal cancer screening for patients age 85 or older without a history of colorectal cancer	USPSTF Lin (2021), USPSTF Whitlock (2008), American Cancer Society (ND)	Ganguli (2021)[1], Park (2021)[2], Kool (2020)[11], Schwartz (2018)[5], Carter (2017)[6], McAlister (2017)[7]

Definition – Base Case		Codes	Include	Exclude
**Numerator**	Patients that received colon cancer screening, sigmoidoscopy, colonoscopy, barium enema, or blood occult test	ICD-10	Z121	
		BETOS		
		CPT/HCPCS	G0104-G0106, G0120-G0122, G0328, 45330-45345, 45378-45392, 82270	
		MS-DRGs/Drug		
		Time	On claim	
		Age		
		Gender		
		Other		
**Denominator**	Patients age 85 or older and do not have a history of colorectal cancer	ICD-10		C18
		BETOS		
		CPT/HCPCS		
		MS-DRGs/Drug		
		Time	On claim	All past data
		Age	85+	
		Gender		
		Other		

Definition – Sensitivity Analysis		Codes	Include	Exclude
**Numerator**	Patients that received colon cancer screening, sigmoidoscopy, colonoscopy, barium enema, or blood occult test	ICD-10	Z121	
		BETOS		
		CPT/HCPCS	G0104-G0106, G0120-G0122, G0328, 45330-45345, 45378-45392, 82270	
		MS-DRGs/Drug		
		Time	On claim	
		Age		

© 2025 Kim DD et al. *JAMA Health Forum*.

		Gender		
		Other		
**Denominator**	Patients age 75 or older and do not have a history of colorectal cancer	ICD-10		C18
		BETOS		
		CPT/HCPCS		
		MS-DRGs/Drug		
		Time	On claim	All past data
		Age	75+	
		Gender		
		Other		

© 2025 Kim DD et al. *JAMA Health Forum*.

3. Prostate-specific antigen (PSA) test

Recommendation	Clinical Source(s)	Low-Value Study Source(s)
Do not perform prostate-specific antigen (PSA) testing in men age 70 or older without a history of prostate cancer	USPSTF Fenton (2018), USPSTF Lin (2008)	Ganguli (2021)[1], Park (2021)[2], Sanghavi (2021)[3], Schwartz (2018)[5], VBID (2018)[12], Carter (2017)[6], McAlister (2017)[7]

Definition – Base Case		Codes	Include	Exclude
**Numerator**	Patients that received a PSA test	ICD-10		
		BETOS		
		CPT/HCPCS	G0103, 84152, 84153, 84154	
		MS-DRGs/Drug		
		Time	On claim	
		Age		
		Gender		
		Other		
**Denominator**	Men age 70 or older without prostate cancer, elevated PSA, **or** family history of prostate cancer	ICD-10		C61, D07.5, D40.0, R97.2, Z80.42
		BETOS		
		CPT/HCPCS		
		MS-DRGs/Drug		
		Time	On claim	All past data
		Age	70+	
		Gender	Male	
		Other		

Definition – Sensitivity Analysis		Codes	Include	Exclude
**Numerator**	Patients that received a PSA test	ICD-10		
		BETOS		
		CPT/HCPCS	G0103, 84152, 84153, 84154	
		MS-DRGs/Drug		
		Time		
		Age	On claim	
		Gender		
		Other		

© 2025 Kim DD et al. *JAMA Health Forum*.

**Denominator**	Men age 70 or older	ICD-10		
		BETOS		
		CPT/HCPCS		
		MS-DRGs/Drug		
		Time	On claim	
		Age	70+	
		Gender	Male	
		Other		

© 2025 Kim DD et al. *JAMA Health Forum*.

4. Cancer screening in dialysis patients

Recommendation	Clinical Source(s)	Low-Value Study Source(s)
Do not perform routine cancer screening for dialysis patients with limited life expectancy	CW, American Society of Nephrology (2012)	Schwartz (2018)[5], McAlister (2017)[7]

Definition – Base Case		Codes	Include	Exclude
**Numerator**	Patients that received breast, cervix, colon, or prostate cancer screening	ICD-10	Z121, Z123, Z124, Z125	
		BETOS		
		CPT/HCPCS	77057, G0202, G0104-G0106, G0120-G0122, G0328, 45330-45345, 45378-45392, 82270, G0102, G0103, 84152-84154, G0101, G0123, G0124, G0141, G0143, G0144, G0145, G0147, G0148, P3000, P3001, Q0091	
		MS-DRGs/Drug		
		Time	On claim	
		Age		
		Gender		
		Other		
**Denominator**	Patients age 75 years or older and on dialysis	ICD-10	Z49	
		BETOS	P9A, P9B	
		CPT/HCPCS		
		MS-DRGs/Drug		
		Time	All past data	
		Age	75+	
		Gender		
		Other		

No definition for sensitivity analysis

© 2025 Kim DD et al. *JAMA Health Forum*.

5. Bacteriuria screening

Recommendation	Clinical Source(s)	Low-Value Study Source(s)
Do not perform screening for bacteriuria in asymptomatic nonpregnant adults	USPSTF Owens (2019)	Shahzad (2022)[13]

Definition – Base Case		Codes	Include	Exclude
**Numerator**	Patients that received bacteriuria screening	ICD-10		
		BETOS		
		CPT/HCPCS	87086	
		MS-DRGs/Drug		
		Time		
		Age		
		Gender		
		Other		
**Denominator**	Patients without a concurrent (same day) diagnosis for dysuria or urinary frequency	ICD-10		R300, R350
		BETOS		
		CPT/HCPCS		
		MS-DRGs/Drug		
		Time		On claim
		Age		
		Gender		
		Other		

No definition for sensitivity analysis

© 2025 Kim DD et al. *JAMA Health Forum*.

6. Bone mineral density (BMD) test

Recommendation	Clinical Source(s)	Low-Value Study Source(s)
Do not perform bone mineral density testing within 2 years of a prior bone mineral density test for patients with an established osteoporosis diagnosis	CW Canadian Association of Nuclear Medicine (2021), USPSTF Viswanathan (2018), CW American College of Rheumatology (2013), USPSTF Nelson (2011), CW Canadian Cardiovascular Society (ND)	Ganguli (2021), Bouck (2018), Rosenthal (2018), Schwartz (2018), McAlister (2017), Pendrith (2017), Reid (2017), Schpero (2017), Colla (2015)

Definition – Base Case		Codes	Include	Exclude
**Numerator**	Patients that received a bone mineral density test or dual-energy x-ray absorptiometry (DXA)	ICD-10	Z13.820	
		BETOS		
		CPT/HCPCS	76070, 76071, 76078, 76977, 77078-77081, 77083, 78350, 78351	
		MS-DRGs/Drug		
		Time	One test on claim<br>One test in past 2 years	
		Age		
		Gender		
		Other		
**Denominator**	Patients with osteoporosis and without cancer or a fragility fracture	ICD-10	M81	**Cancer:** C00-C96, D00-D09, D37-D49<br>**Fragility fracture:** M48.5, M80, M84.4, M84.5, M84.6, S12, S22, S32, S42, S49, S52, S59, S62
		BETOS		
		CPT/HCPCS		**Fragility fracture:** 27230-27248, 25600, 25605, 25609, 25611, 23600-23630, 23665-23680, 73000, 73010, 73020, 73030, 73040, 73050, 73060, 73070, 73080, 73085, 73090, 73092, 73100, 73110, 73115, 73120, 73130, 73140, 73200, 73201, 73202, 73206, 73218, 73219, 73220, 73221, 73222, 73223, 73225, 73300, 73301, 73302, 73303, 73309
		MS-DRGs/Drug		
		Time	All past data	**Cancer:** All past data

© 2025 Kim DD et al. *JAMA Health Forum*.

				**Fragility fracture**: Within the past 2 years
		Age		
		Gender		
		Other		

Definition – Sensitivity Analysis		Codes	Include	Exclude
**Numerator**	Patients that received a bone mineral density test or dual-energy x-ray absorptiometry (DXA)	ICD-10	Z13.820	
		BETOS		
		CPT/HCPCS	76070, 76071, 76078, 76977, 77078-77081, 77083, 78350, 78351	
		MS-DRGs/Drug		
		Time	One test on claim<br>One test in past 2 years	
		Age		
		Gender		
		Other		
**Denominator**	Patients with osteoporosis	ICD-10	M81	
		BETOS		
		CPT/HCPCS		
		MS-DRGs/Drug		
		Time	All past data	
		Age		
		Gender		
		Other		

© 2025 Kim DD et al. *JAMA Health Forum*.

7. Chronic obstructive pulmonary disease (COPD) screening

Recommendation	Clinical Source(s)	Low-Value Study Source(s)
Do not perform chronic obstructive pulmonary disease screening in asymptomatic adults	USPSTF Mangione (2022)	Shazad (2022)[13]

Definition – Base Case		Codes	Include	Exclude
**Numerator**	Patients that received screening for chronic obstructive pulmonary disease	ICD-10		
		BETOS		
		CPT/HCPCS	94010, 94011, 94012, 94013, 94014, 94015, 94016, 94060, 94070, 94150, 94200, 94375, 94450, 94452, 94453, 94610, 94617, 94618, 94619, 94621, 94640, 94642, 94644, 94645, 94660, 94662, 94664, 94667, 94668, 94669, 94680, 94681, 94690, 94726, 94727, 94728, 94729, 94760, 94761, 94762, 94774, 94775, 94776, 94777, 94780, 94781, 94799	
		MS-DRGs/Drug		
		Time		
		Age		
		Gender		
		Other		
**Denominator**	Patients without a concurrent (same day) diagnosis for chronic obstructive pulmonary disease, asthma, or dyspnea	ICD-10		J41, J42, J43, J44, J45, R060
		BETOS		
		CPT/HCPCS		
		MS-DRGs/Drug		
		Time		On claim
		Age		
		Gender		
		Other		

No definition for sensitivity analysis

© 2025 Kim DD et al. *JAMA Health Forum*.

8. Homocysteine test

Recommendation	Clinical Source(s)	Low-Value Study Source(s)
Do not perform homocysteine testing with no diagnoses of folate or B12 deficiencies		Schwartz (2018)[5], McAlister (2017)[7], Reid (2017)[14]

Definition – Base Case		Codes	Include	Exclude
**Numerator**	Patients that received homocysteine testing	ICD-10		
		BETOS		
		CPT/HCPCS	83090	
		MS-DRGs/Drug		
		Time	On claim	
		Age		
		Gender		
		Other		
**Denominator**	Patients without a diagnosis of folate or B12 deficiencies or prior folate or B12 testing	ICD-10		**Diagnosis:** D51, D52, D64.9, D81.818, D81.819, E53.8, E53.9, E72.1
		BETOS		
		CPT/HCPCS		**Prior testing:** 82746, 82747, 82607
		MS-DRGs/Drug		
		Time		**Diagnosis:** On claim<br>**Prior testing:** All past data
		Age		
		Gender		
		Other		

Definition – Sensitivity Analysis		Codes	Include	Exclude
**Numerator**	Patients that received homocysteine testing	ICD-10		
		BETOS		
		CPT/HCPCS	83090	
		MS-DRGs/Drug		
		Time	On claim	
		Age		
		Gender		

© 2025 Kim DD et al. *JAMA Health Forum*.

		Other		
**Denominator**	Patients without a diagnosis of folate or B12 deficiencies	ICD-10		D51, D52, D64.9, D81.818, D81.819, E53.8, E53.9, E72.1
		BETOS		
		CPT/HCPCS		
		MS-DRGs/Drug		
		Time		On claim
		Age		
		Gender		
		Other		

© 2025 Kim DD et al. *JAMA Health Forum*.

9. Hypercoagulability test

Recommendation	Clinical Source(s)	Low-Value Study Source(s)
Do not perform hypercoagulability test in patients with deep vein thrombosis (DVT) with a known cause	CW Society for Vascular Medicine (2013)	Schwartz (2018)[5], McAlister (2017)[7], Reid (2017)[14]

Definition – Base Case		Codes	Include	Exclude
**Numerator**	Patients that received a hypercoagulability test	ICD-10		
		BETOS		
		CPT/HCPCS	81240, 81241, 83090, 85300, 85303, 85306, 85613, 86147	
		MS-DRGs/Drug		
		Time	On claim	
		Age		
		Gender		
		Other		
**Denominator**	Patients with pulmonary embolism or venous embolism with thrombosis	ICD-10	I26, I82	
		BETOS		
		CPT/HCPCS		
		MS-DRGs/Drug		
		Time	Within the past 90 days	
		Age		
		Gender		
		Other		

No definition for sensitivity analysis

© 2025 Kim DD et al. *JAMA Health Forum*.

10. Parathyroid hormone (PTH) measurement

Recommendation	Clinical Source(s)	Low-Value Study Source(s)
Do not perform parathyroid hormone (PTH) measurement for patients with chronic kidney disease		Schwartz (2018)[5], Reid (2017)[14]

Definition – Base Case		Codes	Include	Exclude
**Numerator**	Patients that received PTH measurement	ICD-10		
		BETOS		
		CPT/HCPCS	83970	
		MS-DRGs/Drug		
		Time	On claim	
		Age		
		Gender		
		Other		
**Denominator**	Patients with chronic kidney disease and not on dialysis and without hypercalcemia diagnosis	ICD-10	I12, I13, N18	**Hypercalcemia:** E83.52
		BETOS		**Dialysis:** P9A, P9B
		CPT/HCPCS		
		MS-DRGs/Drug		
		Time	All past data	**Hypercalcemia:** Within the past 1 year<br>**Dialysis:** Any time before claim or within 30 days after claim
		Age		
		Gender		
		Other		

Definition – Sensitivity Analysis		Codes	Include	Exclude
**Numerator**	Patients that received PTH measurement	ICD-10		
		BETOS		
		CPT/HCPCS	83970	
		MS-DRGs/Drug		
		Time	On claim	
		Age		

© 2025 Kim DD et al. *JAMA Health Forum*.

		Gender		
		Other		
**Denominator**	Patients with chronic kidney disease and not on dialysis	ICD-10	I12, I13, N18	
		BETOS		P9A, P9B
		CPT/HCPCS		
		MS-DRGs/Drug		
		Time	All past data	Any time before claim or within 30 days after claim
		Age		
		Gender		
		Other		

© 2025 Kim DD et al. *JAMA Health Forum*.

11. Total or free T3 level test

Recommendation	Clinical Source(s)	Low-Value Study Source(s)
Do not perform a total or free T3 level in patients with hypothyroidism	CW Endocrine Society (2017/2013)	Sanghavi (2021)[3], Schwartz (2018)[5], Reid (2017)[14], Charlesworth (2016)[15]

Definition – Base Case		Codes	Include	Exclude
**Numerator**	Patients that received total or free T3 testing	ICD-10		
		BETOS		
		CPT/HCPCS	84480, 84481	
		MS-DRGs/Drug		
		Time	On claim	
		Age		
		Gender		
		Other		
**Denominator**	Patients with hypothyroidism	ICD-10	E01.8, E02, E03, E89.0	
		BETOS		
		CPT/HCPCS		
		MS-DRGs/Drug		
		Time	Within the past 1 year	
		Age		
		Gender		
		Other		

No definition for sensitivity analysis

© 2025 Kim DD et al. *JAMA Health Forum*.

12. Vitamin D test

Recommendation	Clinical Source(s)	Low-Value Study Source(s)
Do not perform vitamin D testing in non-high risk patients	USPSTF Kahwati (2021), USPSTF LeFevre (2015), CW American Society for Clinical Pathology (2013), CW Endocrine Society (2013)	Ganguli (2021)[1], Rosenthal (2018)[16], Schwartz (2018)[5], VBID (2018)[12], Mafi (2017)[17], Reid (2017)[14], Schpero (2017)[9], Colla (2015)[10]

Definition – Base Case		Codes	Include	Exclude
**Numerator**	Patients that received a vitamin D test	ICD-10		
		BETOS		
		CPT/HCPCS	82306, 82562	
		MS-DRGs/Drug		
		Time	On claim	
		Age		
		Gender		
		Other		
**Denominator**	Patients without chronic kidney disease, hypercalcemia, chronic conditions, secondary hyperparathyroidism of renal origin, sarcoidosis, TB, other select neoplasms, pregnancy, obesity, diabetes, dialysis, osteoporosis, fragility fractures, or fall/non-traumatic fracture	ICD-10		**Chronic conditions:** B52.0, C44, C50, C56, C64, C65, C67, C90, C92, C93, C94, C95, D45, E08, E09, E10, E11, E13, E20.0, E20.8, E20.9, E21.0- E21.5, E55.0, E55.9, E64.3, E67, E68, E83.51, E83.52, E84, E89.2, G73.7, I12, I13, K50, K51, K52.0, K70.2, K70.30, K70.31, K70.41, K71.11, K72, K74, K75.81, K76.0, K76.2, K76.89, K90.0- K90.4, K90.89, K90.9, K91.2, L40.0-L40.4, L40.50-L40.59, L40.8, L40.9, L41, L94.5, M32, M33, M36.0, M80, M81, M83, M85.9, M88, M89.9, M94.9, N08, N16, N18, N25.1, N25.81, Q78.0, Q78.2, Z13.820, Z13.89, Z49.31, Z49.32, Z79.5, Z79.899, Z91.15, Z98.84, Z99.2<br>**Other risk factors**: A15, A17, A18, A19, B38, B39, C81, C82, C83, C84, C85, C86, C88, C91, C96, D86, E44.0, E83, G40, J63

© 2025 Kim DD et al. *JAMA Health Forum*.

				**Pregnancy/Obesity**: A34, E65, E66, O00, O01, O02, O03, O04, O07, O08, O09, O10, O11, O12, O13, O14, O15, O16, O20, O21, O23, O24, O25, O26, O29, O30, O31, O32, O33, O34, O35, O36, O40, O41, O42, O43, O44, O45, O46, O47, O48, O60, O61, O62, O63, O64, O65, O66, O67, O68, O69, O70, O71, O72, O73, O74, O75, O76, O77, O80, O82, O90, O98, O99, O9A, P50, Z32, Z33, Z34, Z36, Z68<br>**Fragility fracture**: M48.5, M80, M84.4, M84.5, M84.6, S12, S22, S32, S42, S49, S52, S59, S62, Z87.310, Z87.311, Z91.81
		BETOS		
		CPT/HCPCS		**Fragility fracture**: 27230-27248, 25600, 25605, 25609, 25611, 23600-23630, 23665-23680, 73000, 73010, 73020, 73030, 73040, 73050, 73060, 73070, 73080, 73085, 73090, 73092, 73100, 73110, 73115, 73120, 73130, 73140
		MS-DRGs/Drug		
		Time		**Chronic conditions:** All past data<br>**Other risk factors:** Within the past 90 days<br>**Pregnancy or obesity:** On claim<br>**Fragility fracture:** Within the past 1 year
		Age		
		Gender		
		Other		

**Definition – Sensitivity Analysis**		**Codes**	**Include**	**Exclude**
**Numerator**	Patients that received a vitamin D test	ICD-10		
		BETOS		
		CPT/HCPCS	82306, 82562	
		MS-DRGs/Drug		

© 2025 Kim DD et al. *JAMA Health Forum*.

		Time	On claim	
		Age		
		Gender		
		Other		
**Denominator**	Patients without chronic kidney disease, hypercalcemia, chronic conditions, secondary hyperparathyroidism of renal origin, sarcoidosis, TB, other select neoplasms	ICD-10		**Chronic conditions:** B52.0, C44, C50, C56, C64, C65, C67, C90, C92, C93, C94, C95, D45, E08, E09, E10, E11, E13, E20.0, E20.8, E20.9, E21.0- E21.5, E55.0, E55.9, E64.3, E67, E68, E83.51, E83.52, E84, E89.2, G73.7, I12, I13, K50, K51, K52.0, K70.2, K70.30, K70.31, K70.41, K71.11, K72, K74, K75.81, K76.0, K76.2, K76.89, K90.0- K90.4, K90.89, K90.9, K91.2, L40.0-L40.4, L40.50-L40.59, L40.8, L40.9, L41, L94.5, M32, M33, M36.0, M80, M81, M83, M85.9, M88, M89.9, M94.9, N08, N16, N18, N25.1, N25.81, Q78.0, Q78.2, Z13.820, Z13.89, Z49.31, Z49.32, Z79.5, Z79.899, Z91.15, Z98.84, Z99.2<br>**Other risk factors**: A15, A17, A18, A19, B38, B39, C81, C82, C83, C84, C85, C86, C88, C91, C96, D86, E44.0, E83, G40, J63
		BETOS		
		CPT/HCPCS		
		MS-DRGs/Drug		
		Time		**Chronic conditions:** All past data<br>**Other risk factors:** Within the past 90 days
		Age		
		Gender		
		Other		

© 2025 Kim DD et al. *JAMA Health Forum*.

13. Preoperative cardiac stress test

Recommendation	Clinical Source(s)	Low-Value Study Source(s)
Do not perform cardiac stress testing not associated with inpatient or emergency care and occurring within 30 days prior to a low or intermediate risk non-cardiothoracic surgical procedure	CW American College of Cardiology (2021), CW American Society of Anesthesiologists (2013), CW The Society of Thoracic Surgeons (2013), CW Society for Vascular Medicine (2013)	Ganguli (2021)[1], Rosenthal (2018)[16], Schwartz (2018)[5], VBID (2018)[12], McAlister (2017)[7], Reid (2017)[14], Schpero (2017)[9], Charlesworth (2016)[15], Colla (2015)[10]

Definition – Base Case		Codes	Include	Exclude
**Numerator**	Patients that received a stress test, nuclear medicine imaging, cardiac MRI **or** CT angiography **and** was not in an inpatient **or** emergency care setting	ICD-10		
		BETOS		
		CPT/HCPCS	75552-75564, 75574, 78451-78454, 78460, 78461, 78464, 78465, 78472, 78473, 78481, 78483, 78491, 78492, 93015-93018, 93024, 93350, 93351, 93352, 0146T, 0147T, 0148T, 0149T	**All inpatient**<br>**Emergency care:** 99281-99285, 99288
		MS-DRGs/Drug		
		Time	Within the past 30 days	On same claim as test
		Age		
		Gender		
		Other		
**Denominator**	Patients undergoing low or intermediate risk non-cardiothoracic surgical procedure	ICD-10		
		BETOS	P1x, P3D, P4A, P4B, P4C, P5C, P5D, P8A, P8G	
		CPT/HCPCS	19120, 19125, 47562, 47563, 49560, 58558	
		MS-DRGs/Drug		
		Time	On claim	
		Age		
		Gender		
		Other		

Definition – Sensitivity Analysis		Codes	Include	Exclude
**Numerator**		ICD-10		

© 2025 Kim DD et al. *JAMA Health Forum*.

	Patients that received a stress test, nuclear medicine imaging, cardiac MRI **or** CT angiography **and** was not in an inpatient **or** emergency care setting	BETOS		
		CPT/HCPCS	75552-75564, 75574, 78451-78454, 78460, 78461, 78464, 78465, 78472, 78473, 78481, 78483, 78491, 78492, 93015-93018, 93024, 93350, 93351, 93352, 0146T, 0147T, 0148T, 0149T	
		MS-DRGs/Drug		
		Time	Within the past 30 days	
		Age		
		Gender		
		Other		
**Denominator**	Patients undergoing low or intermediate risk non-cardiothoracic surgical procedure and without warranted diagnosis.<br><br>Warranted diagnosis includes: cardiac conditions, respiratory conditions except acute respiratory infections, acute central nervous system conditions except headaches, all thoracic and non-thoracic arterial conditions except peripheral venous conditions, malignant hypertension, end stage renal disease and acute renal disease, abdominal pain, cancer, drug toxicity or poisoning, exertional stress, or fatigue	ICD-10		**Warranted diagnoses**: A31.0, A02.1, A22.7, A26.7, A32.7, A40, A41, A42.7, A49.01, A49.02, A54.86, A69.2, B20, B25.2, B37.7, B95.61, B95.62, B97.10, B97.89, C18.9, C20, C25.9, C34.1, C34.8, C34.9, C43.9, C44.2, C44.3, C44.4, C44.7, C44.9, C45.9, C50.4, C50.1, C50.9, C56, C61, C64, C73, C78.0, C79.5, C80.1, C82.5, C84.9, C84.A0, C84.A9, C84.Z0, C84.Z9, C85, C86.4, C90.00, C91.10, D03.9, D05, D07.5, D14.3, D32.0, D32.9, D37, D38.1, D40.0, D41, D45, D46, D47, D48.5, D48.6, D49 – D53, D59.9, D60, D61, D62, D63.1, D63.8, D64, D65, D68.3, D69, D70.9, D71, D72.8, D75, D77, D78, D86, D89.2, D89.8, E06.0, E11, E13.00, E13.01, E13.10, E20.1, E27.1, E27.2, E27.3, E27.4, E36.0, E36.1, E46, E64.0, E72.1, E79.0, E83.1, E83.3, E83.4, E83.5, E86.0, E86.9, E87.0, E87.1, E87.2, E87.5, E87.6, E87.7, E89.6, E89.82, F05, F07.0, F10.1, F10.20, F11.2, F18.1, F18.90, F19.10, F19.20, F20.3, F20.9, F28, F29, F31.7, F31.9, F32.9, F33.2, F33.40, F33.9, F34.1, F41, F43.23, F52.8, F55, G35, G44.1, G45, G46,

© 2025 Kim DD et al. *JAMA Health Forum*.

				G89.18, G91, G93.3, G93.4, G97.3, G97.4, G97.6, H53.04, H53.7, H53.8, H59.1, H59.2, H59.3, H81.1, H81.39, H95.2, H95.3, H95.5, I05, I06.0, I07, I08.0, I08.9, I10, I11, I12, I20, I21, I22.2, I24-I27, I31.3, I31.9, I33.0, I34-I37, I38, I42, I44-I51, I52, I60, I61, I62.0, I63, I65, I66, I67, I69, I70, I71.2, I71.4, I71.9, I73.0, I74.3, I74.4, I77, I79.0, I82.2, I82.4, I82.9, I87.8, I95.1, I95.9, I97.1, I97.4, I97.5, I97.6, I97.7, I97.8, I99.8, J02.8, J02.9, J06.9, J10.0, J10.1, J11.0, J11.1, J12.0, J12.1, J12.9, J13, J15.8, J15.9, J18, J20, J22, J39.8, J40, J41.0, J43, J44, J45, J47, J61, J69.0, J80, J81, J82, J84, J86.9, J90, J91, J92, J93.1, J94, J95, J96, J98, K20, K21, K44.9, K51.9, K59.31, K62.5, K68.11, K85, K90.0, K91.6, K91.7, K91.87, K92, L40, L76, L93, M00, M04.9, M05.2, M05.3, M05.4, M05.5, M05.6, M05.7, M05.8, M06, M13.0, M25.4, M25.5, M25.7, M30.1, M31.3, M31.5, M31.6, M32, M34, M35, M40, M41.4, M41.5, M46.2, M48.5, M51.34, M51.35, M53.8, M54.0, M54.1, M54.6, M54.8, M54.9, M60.8, M60.9, M62.8, M75.3, M75.4, M75.8, M75.9, M79.0, M79.1, M79.6, M79.7, M79.89, M80.08, M80.88, M84.48, M84.58, M84.68, M86.08, M86.18, M86.28, M86.9, M95.4, M96.8, M99.8, N17.9, N18.6, N60.1, N63, N64.4, N64.5, N95.0, N99.6, N99.7, N99.84, Q20.9, Q21.1, Q23.3, Q23.8, Q23.9, Q24.5, Q24.8, Q24.9, Q25.29, Q25.3, Q25.4, Q25.8, Q25.9, Q33.9, Q79.0, Q79.1, R00, R01, R04, R05, R06, R07, R09, R10, R13, R18.8, R19, R22, R23.3, R40.0, R40.1,

© 2025 Kim DD et al. *JAMA Health Forum*.

				R40.4, R41.0, R41.1, R41.2, R41.3, R41.82, R42, R45.0, R45.3, R45.4, R45.86, R45.87, R45.89, R50, R51, R53, R55, R57.1, R57.8, R58, R59, R60, R63.4, R68.0, R68.83, R68.89, R70.0, R71.8, R73, R76.1, R78.7, R78.8, R79.0, R79.8, R79.9, R90.0, R91, R92, R93.1, R93.8, R94.2, R94.3, R97.8, S00, S06, S09, S10, S12, S14, S16, S19, S20.2, S22, S27, S29, S32, S39, S40, S42, S43, S46, S49, T07, T14, T36-T50, T80.0, T81.4, T81.7, T82, T86.2, T86.3, T86.8, T88.5, T88.8, V00, V89, W00, W01, W04, W18, W19, X58, Y83.8, Z03.89, Z04.1, Z04.3, Z09, Z11.1, Z13.0, Z20.1, Z45, Z46.82, Z47.2, Z48.2, Z49.31, Z51.1, Z57.8, Z77.090, Z79.3, Z79.84, Z79.891, Z79.899, Z85.038, Z85.118, Z85.3, Z85.46, Z85.51, Z85.828, Z86.11, Z86.7, Z91.81, Z94.0, Z94.1, Z94.2, Z94.3, Z94.4, Z95.0, Z95.1, Z95.2, Z95.5, Z95.810, Z98.61, Z98.890, Z99.2
		BETOS	P1x, P3D, P4A, P4B, P4C, P5C, P5D, P8A, P8G	
		CPT/HCPCS	19120, 19125, 47562, 47563, 49560, 58558	
		MS-DRGs/Drug		
		Time	On claim	Within the past 30 days
		Age		
		Gender		
		Other		

© 2025 Kim DD et al. *JAMA Health Forum*.

14. Preoperative chest radiography

Recommendation	Clinical Source(s)	Low-Value Study Source(s)
Do not perform a chest X-ray not associated with inpatient or emergency care and occurring within 30 days prior to a low or intermediate risk non-cardiothoracic surgical procedure	CW American College of Cardiology (2021), CW American College of Radiology (2017/2013), CW American College of Surgeons (2013), CW American College of Physicians (2012), CW American Society of Anesthesiologists (2012), CW American Society of Nuclear Cardiology (2012)	Ganguli (2021)[1], Koehlmoos (2019)[18], Oakes (2019)[19], Schwartz (2018)[5], VBID (2018)[12], Carter (2017)[6], Reid (2017)[14], Schpero (2017)[9], Charlesworth (2016)[15], Colla (2015)[10], Segal (2014)[20]

Definition – Base Case		Codes	Include	Exclude
**Numerator**	Patients that received a chest x-ray and was not in an inpatient or emergency care setting	ICD-10		
		BETOS		
		CPT/HCPCS	71045, 71046, 71047, 71048, 71010, 71015, 71020, 71021, 71022, 71030, 71035	All inpatient<br>Emergency care: 99281-99285, 99288
		MS-DRGs/Drug		
		Time	Within the past 30 days	On same claim as test
		Age		
		Gender		
		Other		
**Denominator**	Patients undergoing low or intermediate risk non-cardiothoracic surgical procedure	ICD-10		
		BETOS	P1x, P3D, P4A, P4B, P4C, P5C, P5D, P8A, P8G	
		CPT/HCPCS	19120, 19125, 47562, 47563, 49560, 58558	
		MS-DRGs/Drug		
		Time	On claim	
		Age		
		Gender		
		Other		

Definition – Sensitivity Analysis		Codes	Include	Exclude
**Numerator**		ICD-10		

© 2025 Kim DD et al. *JAMA Health Forum*.

	Patients that received a chest x-ray	BETOS		
		CPT/HCPCS	71045, 71046, 71047, 71048, 71010, 71015, 71020, 71021, 71022, 71030, 71035	
		MS-DRGs/Drug		
		Time	Within the past 30 days	
		Age		
		Gender		
		Other		
**Denominator**	Patients undergoing low or intermediate risk non-cardiothoracic surgical procedure and without warranted diagnosis.<br><br>Warranted diagnosis includes: cardiac conditions, respiratory conditions except acute respiratory infections, acute central nervous system conditions except headaches, all thoracic and non-thoracic arterial conditions except peripheral venous conditions, malignant hypertension, end stage renal disease and acute renal disease, abdominal pain, cancer, drug toxicity or poisoning, exertional stress, or fatigue	ICD-10		See Warranted Diagnosis codes from 13. Preoperative cardiac stress test
		BETOS	P1x, P3D, P4A, P4B, P4C, P5C, P5D, P8A, P8G	
		CPT/HCPCS	19120, 19125, 47562, 47563, 49560, 58558	
		MS-DRGs/Drug		
		Time	On claim	Within the past 30 days
		Age		
		Gender		
		Other		

© 2025 Kim DD et al. *JAMA Health Forum*.

15. Preoperative echocardiography

Recommendation	Clinical Source(s)	Low-Value Study Source(s)
Do not perform an echocardiogram not associated with inpatient or emergency care and occurring within 30 days prior to a low or intermediate risk non-cardiothoracic surgical procedure	CW American Society of Anesthesiologists (2013), CW American Society of Echocardiography (2013)	Ganguli (2021)[1], Rosenthal (2018)[16], Schwartz (2018)[5], VBID (2018)[12], Carter (2017)[6], Reid (2017)[14], Schpero (2017)[9], Charlesworth (2016)[15], Colla (2015)[10]

Definition – Base Case		Codes	Include	Exclude
**Numerator**	Patients that received an echocardiogram and was not in an inpatient or emergency care setting	ICD-10		
		BETOS		
		CPT/HCPCS	93303, 93304, 93306-93308, 93312, 93315, 93318	All inpatient<br>Emergency care: 99281-99285, 99288
		MS-DRGs/Drug		
		Time	Within the past 30 days	On same claim as test
		Age		
		Gender		
		Other		
**Denominator**	Patients undergoing low or intermediate risk non-cardiothoracic surgical procedure	ICD-10		
		BETOS	P1x, P3D, P4A, P4B, P4C, P5C, P5D, P8A, P8G	
		CPT/HCPCS	19120, 19125, 47562, 47563, 49560, 58558	
		MS-DRGs/Drug		
		Time	On claim	
		Age		
		Gender		
		Other		

Definition – Sensitivity Analysis		Codes	Include	Exclude
**Numerator**	Patients that received an echocardiogram	ICD-10		
		BETOS		

© 2025 Kim DD et al. *JAMA Health Forum*.

		CPT/HCPCS	93303, 93304, 93306-93308, 93312, 93315, 93318	
		MS-DRGs/Drug		
		Time	Within the past 30 days	
		Age		
		Gender		
		Other		
**Denominator**	Patients undergoing low or intermediate risk non-cardiothoracic surgical procedure and without warranted diagnosis.<br><br>Warranted diagnosis includes: cardiac conditions, respiratory conditions except acute respiratory infections, acute central nervous system conditions except headaches, all thoracic and non-thoracic arterial conditions except peripheral venous conditions, malignant hypertension, end stage renal disease and acute renal disease, abdominal pain, cancer, drug toxicity or poisoning, exertional stress, or fatigue	ICD-10		See Warranted Diagnosis codes from 13. Preoperative cardiac stress test
		BETOS	P1x, P3D, P4A, P4B, P4C, P5C, P5D, P8A, P8G	
		CPT/HCPCS	19120, 19125, 47562, 47563, 49560, 58558	
		MS-DRGs/Drug		
		Time	On claim	Within the past 30 days
		Age		
		Gender		
		Other		

© 2025 Kim DD et al. *JAMA Health Forum*.

16. Preoperative pulmonary function test (PFT)

Recommendation	Clinical Source(s)	Low-Value Study Source(s)
Do not perform a pulmonary function test (PFT) not associated with inpatient or emergency care and occurring within 30 days prior to a low or intermediate risk non-cardiothoracic surgical procedure	CW The Society of Thoracic Surgeons (2021/2013), CW American Society of Anesthesiologists (2012)	Ganguli (2021)[1], Schwartz (2018)[5], VBID (2018)[12], Reid (2017)[14], Schpero (2017)[9], Charlesworth (2016)[15], Colla (2015)[10]

Definition – Base Case		Codes	Include	Exclude
**Numerator**	Patients that received pulmonary function testing and was not in an inpatient or emergency care setting	ICD-10		
		BETOS		
		CPT/HCPCS	94010	All inpatient<br>Emergency care: 99281-99285, 99288
		MS-DRGs/Drug		
		Time	Within the past 30 days	On the same claim as test
		Age		
		Gender		
		Other		
**Denominator**	Patients undergoing low or intermediate risk non-cardiothoracic surgical procedure	ICD-10		
		BETOS	P1x, P3D, P4A, P4B, P4C, P5C, P5D, P8A, P8G	
		CPT/HCPCS	19120, 19125, 47562, 47563, 49560, 58558	
		MS-DRGs/Drug		
		Time	On claim	
		Age		
		Gender		
		Other		

Definition – Sensitivity Analysis		Codes	Include	Exclude
**Numerator**	Patients that received pulmonary function testing	ICD-10		
		BETOS		
		CPT/HCPCS	94010	

© 2025 Kim DD et al. *JAMA Health Forum*.

		MS-DRGs/Drug		
		Time	Within the past 30 days	
		Age		
		Gender		
		Other		
**Denominator**	Patients undergoing low or intermediate risk non-cardiothoracic surgical procedure and without warranted diagnosis.<br><br>Warranted diagnosis includes: cardiac conditions, respiratory conditions except acute respiratory infections, acute central nervous system conditions except headaches, all thoracic and non-thoracic arterial conditions except peripheral venous conditions, malignant hypertension, end stage renal disease and acute renal disease, abdominal pain, cancer, drug toxicity or poisoning, exertional stress, or fatigue	ICD-10		See Warranted Diagnosis codes from 13. Preoperative cardiac stress test
		BETOS		
		CPT/HCPCS		
		MS-DRGs/Drug		
		Time		Within the past 30 days
		Age		
		Gender		
		Other		

© 2025 Kim DD et al. *JAMA Health Forum*.

17. Preoperative advanced cardiac imaging for cataract surgery

Recommendation	Clinical Source(s)	Low-Value Study Source(s)
Do not perform advanced cardiac imaging not associated with a warranted diagnosis and occurring within 30 days prior to cataract surgery	CW American College of Cardiology (2021), CW Society of General Internal Medicine (2017/2013), CW American Academy of Ophthalmology (2013)	Colla (2015)[10]

Definition – Base Case		Codes	Include	Exclude
**Numerator**	Patients that received advanced cardiac imaging (CT, MRI, PET)	ICD-10		
		BETOS		
		CPT/HCPCS	0144T, 0145T, 0146T, 0147T, 0148T, 0149T, 0150T, 75552, 75553, 75554, 75555, 75556, 75557, 75558, 75559, 75561, 75562, 75565, 75571, 75572, 75573, 75574, 78451, 78452, 78453, 78454, 78460, 78461, 78464, 78465, 78478, 78480, 78459, 78481, 78483, 78491, 78492, 78494, 78496, 78499	
		MS-DRGs/Drug		
		Time	Within the past 30 days	
		Age		
		Gender		
		Other		
**Denominator**	Patients that received cataract surgery and without a warranted diagnosis<br><br>Warranted diagnoses: proximal fractures, pulmonary and chest conditions, esophageal conditions except mild and chronic, cardiac conditions, end stage renal disease and dialysis, infections including sepsis but not urinary tract infections or pneumonia, anemia and	ICD-10		C00-C97, D00-D48, G00-G13, G35-G37, G40, G41, G45, G46, G47, G90-G99, I05-I15, I20-I52, I70-I79, I95-I99, J40-J47, J60-J70, J80-J86, J90-J99, N10-N19, R10, R53, T36-T65, X40-X57, Y10-Y19
		BETOS	P4B	
		CPT/HCPCS		
		MS-DRGs/Drug		
		Time	On claim	Within the past 30 days
		Age		
		Gender		
		Other		

© 2025 Kim DD et al. *JAMA Health Forum*.

	bleeding conditions except microscopic hematuria, abdominal conditions or pain but not benign tumor of the colon, psychiatric conditions, acute central nervous system conditions and seizure or epilepsy conditions but not chronic conditions or migraine, drug toxicity and poisoning, transplants, malignant hypertension, cancer, conditions involving arterial and pulmonary emboli but not peripheral deep vein thrombosis, falls, autoimmune conditions, pain in thorax or shoulder but not specific shoulder conditions			

No definition for sensitivity analysis

© 2025 Kim DD et al. *JAMA Health Forum*.

18. Preoperative cardiac stress test for cataract surgery

Recommendation	Clinical Source(s)	Low-Value Study Source(s)
Do not perform cardiac stress test not associated with a warranted diagnosis and occurring within 30 days prior to cataract surgery	CW American College of Cardiology (2021), CW Society of General Internal Medicine (2017/2013), CW American Academy of Ophthalmology (2013)	Colla (2015)[10]

Definition – Base Case		Codes	Include	Exclude
**Numerator**	Patients that received cardiac stress test	ICD-10		
		BETOS		
		CPT/HCPCS	75560, 75563, 75564, 93015, 93016, 93017, 93018, 93024, 93350, 93351, 93352	
		MS-DRGs/Drug		
		Time	Within the past 30 days	
		Age		
		Gender		
		Other		
**Denominator**	Patients that received cataract surgery and without a warranted diagnosis<br><br>Warranted diagnoses: proximal fractures, pulmonary and chest conditions, esophageal conditions except mild and chronic, cardiac conditions, end stage renal disease and dialysis, infections including sepsis but not urinary tract infections or pneumonia, anemia and bleeding conditions except microscopic hematuria, abdominal conditions or pain but not benign tumor of the	ICD-10		C00-C97, D00-D48, G00-G13, G35-G37, G40, G41, G45, G46, G47, G90-G99, I05-I15, I20-I52, I70-I79, I95-I99, J40-J47, J60-J70, J80-J86, J90-J99, N10-N19, R10, R53, T36-T65, X40-X57, Y10-Y19
		BETOS	P4B	
		CPT/HCPCS		
		MS-DRGs/Drug		
		Time	On claim	Within the past 30 days
		Age		
		Gender		
		Other		

© 2025 Kim DD et al. *JAMA Health Forum*.

	colon, psychiatric conditions, acute central nervous system conditions and seizure or epilepsy conditions but not chronic conditions or migraine, drug toxicity and poisoning, transplants, malignant hypertension, cancer, conditions involving arterial and pulmonary emboli but not peripheral deep vein thrombosis, falls, autoimmune conditions, pain in thorax or shoulder but not specific shoulder conditions			

No definition for sensitivity analysis

© 2025 Kim DD et al. *JAMA Health Forum*.

19. Preoperative chest radiography for cataract surgery

Recommendation	Clinical Source(s)	Low-Value Study Source(s)
Do not perform chest x-ray not associated with a warranted diagnosis and occurring within 30 days prior to cataract surgery	CW American College of Cardiology (2021), CW Society of General Internal Medicine (2017/2013), CW American Academy of Ophthalmology (2013)	Colla (2015)[10]

Definition – Base Case		Codes	Include	Exclude
**Numerator**	Patients that received chest x-ray	ICD-10		
		BETOS		
		CPT/HCPCS	71045, 71046, 71047, 71048	
		MS-DRGs/Drug		
		Time	Within the past 30 days	
		Age		
		Gender		
		Other		
**Denominator**	Patients that received cataract surgery and without a warranted diagnosis<br><br>Warranted diagnoses: proximal fractures, pulmonary and chest conditions, esophageal conditions except mild and chronic, cardiac conditions, end stage renal disease and dialysis, infections including sepsis but not urinary tract infections or pneumonia, anemia and bleeding conditions except microscopic hematuria, abdominal conditions or pain but not benign tumor of the colon, psychiatric conditions, acute central nervous	ICD-10		C00-C97, D00-D48, G00-G13, G35-G37, G40, G41, G45, G46, G47, G90-G99, I05-I15, I20-I52, I70-I79, I95-I99, J40-J47, J60-J70, J80-J86, J90-J99, N10-N19, R10, R53, T36-T65, X40-X57, Y10-Y19
		BETOS	P4B	
		CPT/HCPCS		
		MS-DRGs/Drug		
		Time	On claim	Within the past 30 days
		Age		
		Gender		
		Other		

© 2025 Kim DD et al. *JAMA Health Forum*.

	system conditions and seizure or epilepsy conditions but not chronic conditions or migraine, drug toxicity and poisoning, transplants, malignant hypertension, cancer, conditions involving arterial and pulmonary emboli but not peripheral deep vein thrombosis, falls, autoimmune conditions, pain in thorax or shoulder but not specific shoulder conditions			

No definition for sensitivity analysis

© 2025 Kim DD et al. *JAMA Health Forum*.

20. Preoperative echocardiogram for cataract surgery

Recommendation	Clinical Source(s)	Low-Value Study Source(s)
Do not perform echocardiogram not associated with a warranted diagnosis and occurring within 30 days prior to cataract surgery	CW American College of Cardiology (2021), CW Society of General Internal Medicine (2017/2013), CW American Academy of Ophthalmology (2013)	Colla (2015)[10]

Definition – Base Case		Codes	Include	Exclude
**Numerator**	Patients that received echocardiogram	ICD-10		
		BETOS		
		CPT/HCPCS	93303, 93304, 93306, 93307, 93308, 93320, 93321, 93325	
		MS-DRGs/Drug		
		Time	Within the past 30 days	
		Age		
		Gender		
		Other		
**Denominator**	Patients that received cataract surgery and without a warranted diagnosis<br><br>Warranted diagnoses: proximal fractures, pulmonary and chest conditions, esophageal conditions except mild and chronic, cardiac conditions, end stage renal disease and dialysis, infections including sepsis but not urinary tract infections or pneumonia, anemia and bleeding conditions except microscopic hematuria, abdominal conditions or pain but not benign tumor of the colon, psychiatric conditions, acute central nervous	ICD-10		C00-C97, D00-D48, G00-G13, G35-G37, G40, G41, G45, G46, G47, G90-G99, I05-I15, I20-I52, I70-I79, I95-I99, J40-J47, J60-J70, J80-J86, J90-J99, N10-N19, R10, R53, T36-T65, X40-X57, Y10-Y19
		BETOS	P4B	
		CPT/HCPCS		
		MS-DRGs/Drug		
		Time	On claim	Within the past 30 days
		Age		
		Gender		
		Other		

© 2025 Kim DD et al. *JAMA Health Forum*.

	system conditions and seizure or epilepsy conditions but not chronic conditions or migraine, drug toxicity and poisoning, transplants, malignant hypertension, cancer, conditions involving arterial and pulmonary emboli but not peripheral deep vein thrombosis, falls, autoimmune conditions, pain in thorax or shoulder but not specific shoulder conditions			

No definition for sensitivity analysis

© 2025 Kim DD et al. *JAMA Health Forum*.

21. Preoperative electrocardiogram for cataract surgery

Recommendation	Clinical Source(s)	Low-Value Study Source(s)
Do not perform electrocardiogram not associated with a warranted diagnosis and occurring within 30 days prior to cataract surgery	CW American College of Cardiology (2021), CW Society of General Internal Medicine (2017/2013), CW American Academy of Ophthalmology (2013)	Colla (2015)[10]

Definition – Base Case		Codes	Include	Exclude
**Numerator**	Patients that received electrocardiogram	ICD-10		
		BETOS		
		CPT/HCPCS	3120F, 93000, 93005, 93010, G0366, G0367, G0368, G0403, G0404, G0405	
		MS-DRGs/Drug		
		Time	Within the past 30 days	
		Age		
		Gender		
		Other		
**Denominator**	Patients that received cataract surgery and without a warranted diagnosis<br><br>Warranted diagnoses: proximal fractures, pulmonary and chest conditions, esophageal conditions except mild and chronic, cardiac conditions, end stage renal disease and dialysis, infections including sepsis but not urinary tract infections or pneumonia, anemia and bleeding conditions except microscopic hematuria, abdominal conditions or pain but not benign tumor of the colon, psychiatric conditions, acute central nervous	ICD-10		C00-C97, D00-D48, G00-G13, G35-G37, G40, G41, G45, G46, G47, G90-G99, I05-I15, I20-I52, I70-I79, I95-I99, J40-J47, J60-J70, J80-J86, J90-J99, N10-N19, R10, R53, T36-T65, X40-X57, Y10-Y19
		BETOS	P4B	
		CPT/HCPCS		
		MS-DRGs/Drug		
		Time	On claim	Within the past 30 days
		Age		
		Gender		
		Other		

© 2025 Kim DD et al. *JAMA Health Forum*.

	system conditions and seizure or epilepsy conditions but not chronic conditions or migraine, drug toxicity and poisoning, transplants, malignant hypertension, cancer, conditions involving arterial and pulmonary emboli but not peripheral deep vein thrombosis, falls, autoimmune conditions, pain in thorax or shoulder but not specific shoulder conditions			

No definition for sensitivity analysis

© 2025 Kim DD et al. *JAMA Health Forum*.

22. Carotid imaging for asymptomatic patients

Recommendation	Clinical Source(s)	Low-Value Study Source(s)
Do not perform carotid imaging not associated with inpatient or emergency care for patients without stroke, TIA, or focal neurological symptoms	USPSTF Guirguis-Blake et al (2021), USPSTF Jonas (2014), USPSTF Wolff (2007)	Koehlmoos (2019)[18], Oakes (2019)[19], Schwartz (2018)[5], McAlister (2017)[7], Reid (2017)[14], Segal (2014)[20]

Definition – Base Case		Codes	Include	Exclude
**Numerator**	Patients that received carotid imaging **and** was not in an inpatient **or** emergency care setting	ICD-10		
		BETOS		
		CPT/HCPCS	70498, 70547-70549, 93880, 93882, 3100F	All inpatient<br>Emergency care: 99281-99285, 99288
		MS-DRGs/Drug		
		Time	On claim	On claim
		Age		
		Gender		
		Other		
**Denominator**	Patients without stroke/TIA, retinal vascular occlusion/ischemia, or nervous and musculoskeletal symptoms	ICD-10		G45, G46.0, G46.1, G46.2, G81.9, G97.31, G97.32, H34, H35.82, I60, I61, I63, I66, I65.2, I67.2, I67.841, I67.848, I67.89, I97.8, R09.8, R20, R22.0, R22.1, R25, R26, R27, R29, R41.4, R43, R47, R55, R68.3, R90.0, Z86.73
		BETOS		
		CPT/HCPCS		
		MS-DRGs/Drug		
		Time		On claim
		Age		
		Gender		
		Other		

Definition – Sensitivity Analysis		Codes	Include	Exclude
**Numerator**		ICD-10		

© 2025 Kim DD et al. *JAMA Health Forum*.

	Patients that received carotid imaging **and** was not in an inpatient **or** emergency care setting	BETOS		
		CPT/HCPCS	70498, 70547-70549, 93880, 93882, 3100F	All inpatient<br>Emergency care: 99281-99285, 99288
		MS-DRGs/Drug		
		Time	On claim	On claim
		Age		
		Gender		
		Other		
**Denominator**	Patients without stroke/TIA, retinal vascular occlusion/ischemia, or nervous and musculoskeletal symptoms	ICD-10		G45, G46.0, G46.1, G46.2, G97.31, G97.32, H34, H35.82, I60, I61, I63, I66, I67.841, I67.848, I67.89, I97.8, R20, R25, R26, R27, R29, R41.4, R43, R47, R55, R68.3, Z86.73
		BETOS		
		CPT/HCPCS		
		MS-DRGs/Drug		
		Time		On claim
		Age		
		Gender		
		Other		

© 2025 Kim DD et al. *JAMA Health Forum*.

23. Carotid imaging for syncope

Recommendation	Clinical Source(s)	Low-Value Study Source(s)
Do not perform imaging of the carotid arteries for simply syncope without other neurologic symptoms	CW American Academy of Neurology (2013)	Chalmers (2021)[21], Ganguli (2021)[1], Schwartz (2018)[5], McAlister (2017)[7], Reid (2017)[14]

Definition – Base Case		Codes	Include	Exclude
**Numerator**	Patients that received carotid imaging	ICD-10		
		BETOS		
		CPT/HCPCS	36222, 36223, 36224, 70498, 70547-70549, 93880, 93882, 3100F	
		MS-DRGs/Drug		
		Time	On claim	
		Age		
		Gender		
		Other		
**Denominator**	Patients with syncope and without other neurologic symptoms and no previous syncope diagnosis within 2 years before imaging<br>Other neurologic symptoms: without stroke or TIA, history of stroke or TIA, retinal vascular occlusion or ischemia, or nervous or musculoskeletal symptoms	ICD-10	R55, T67.1XXA	**Syncope:** R55, T67.1XXA<br>**Other neurologic symptoms**: G45, G46.0, G46.1, G46.2, G97.3, H34, H35.82, I60, I61, I63, I66, I67.84, I67.89, I97.81, I97.82, R20, R25, R26, R27, R29, R41.4, R43, R47, R68.3, Z86.73
		BETOS		
		CPT/HCPCS		
		MS-DRGs/Drug		
		Time	Within the past 2 weeks	**Syncope:** Within the past 2 years<br>**Other neurologic symptoms:** All past data
		Age		
		Gender		
		Other		

Definition – Sensitivity Analysis		Codes	Include	Exclude
**Numerator**		ICD-10		

© 2025 Kim DD et al. *JAMA Health Forum*.

	Patients that received carotid imaging	BETOS		
		CPT/HCPCS	36222, 36223, 36224, 70498, 70547-70549, 93880, 93882, 3100F	
		MS-DRGs/Drug		
		Time	On claim	
		Age		
		Gender		
		Other		
**Denominator**	Patients with syncope and without other neurologic symptoms<br>Other neurologic symptoms: without stroke or TIA, history of stroke or TIA, retinal vascular occlusion or ischemia, or nervous or musculoskeletal symptoms	ICD-10	R55, T67.1XXA	G45, G46.0, G46.1, G46.2, G97.3, H34, H35.82, I60, I61, I63, I66, I67.84, I67.89, I97.81, I97.82, R20, R25, R26, R27, R29, R41.4, R43, R47, R68.3, Z86.73
		BETOS		
		CPT/HCPCS		
		MS-DRGs/Drug		
		Time	Within the past 2 weeks	All past data
		Age		
		Gender		
		Other		

© 2025 Kim DD et al. *JAMA Health Forum*.

24. CT of sinuses for rhinosinusitis

Recommendation	Clinical Source(s)	Low-Value Study Source(s)
Do not perform maxillofacial CT study with a diagnosis of sinusitis and no other complications	CW American Academy of Allergy, Asthma & Immunology (2021/2012)	Ganguli (2021)[1], Koehlmoos (2019)[18], Oakes (2019)[19], Schwartz (2018)[5], Barnett (2017)[22], Carter (2017)[6], Charlesworth (2016)[15], Segal (2014)[20]

Definition – Base Case		Codes	Include	Exclude
**Numerator**	Patients that received a CT of maxillofacial area	ICD-10		
		BETOS		
		CPT/HCPCS	70486, 70487, 70488	
		MS-DRGs/Drug		
		Time	On claim	
		Age		
		Gender		
		Other		
**Denominator**	Patients with sinusitis and with no other related complications and with no prior sinusitis diagnosis<br>Other related complications: complications of sinusitis, immune deficiencies, nasal polyps, head/face trauma	ICD-10	J01, J32	**Other related complications:** B20, B97.35, D80, D81.0, D81.1, D81.2, D81.4, D81.6, D81.7, D81.89, D81.9, D82-D84, D89.3, D89.4, D89.8, E84, H00, H01, H05.00, J33, L08.89, M35.9, S00-S10, S16, S19<br>**Prior sinusitis diagnosis:** J01, J32
		BETOS		
		CPT/HCPCS		
		MS-DRGs/Drug		
		Time	On claim	**Other related complications:** On claim<br>**Prior sinusitis diagnosis:** Within 30 to 365 days before claim
		Age		
		Gender		
		Other		

Definition – Sensitivity Analysis		Codes	Include	Exclude

© 2025 Kim DD et al. *JAMA Health Forum*.

**Numerator**	Patients that received a CT of maxillofacial area	ICD-10		
		BETOS		
		CPT/HCPCS	70486, 70487, 70488	
		MS-DRGs/Drug		
		Time	On claim	
		Age		
		Gender		
		Other		
**Denominator**	Patients with sinusitis and with no other related complications and with no prior sinusitis diagnosis<br>Other related complications: complications of sinusitis, immune deficiencies, nasal polyps, head/face trauma	ICD-10	J01, J32	**Other related complications**: B20, B97.35, D80, D81.0, D81.1, D81.2, D81.4, D81.6, D81.7, D81.89, D81.9, D82-D84, D89.3, D89.4, D89.8, E84, H00, H01, H05.00, J33, L08.89, M35.9, S00-S10, S16, S19<br>**Prior sinusitis diagnosis**: J01, J32
		BETOS		
		CPT/HCPCS		
		MS-DRGs/Drug		
		Time	Within the past 3 months	**Other related complications**: On claim<br>**Prior sinusitis diagnosis:** Within 3 to 12 months before claim
		Age		
		Gender		
		Other		

© 2025 Kim DD et al. *JAMA Health Forum*.

25. Electroencephalogram (EEG) for headache

Recommendation	Clinical Source(s)	Low-Value Study Source(s)
Do not perform an EEG for headache diagnosis without epilepsy or convulsions	CW American Academy of Neurology (2013)	Chalmers (2021)[21], Ganguli (2021)[1], Schwartz (2018)[5], Reid (2017)[14], Charlesworth (2016)[15]

Definition – Base Case		Codes	Include	Exclude
**Numerator**	Patients that received an electroencephalogram (EEG)	ICD-10		
		BETOS		
		CPT/HCPCS	95812, 95813, 95816, 95819, 95822, 95827, 95830, 95957	
		MS-DRGs/Drug		
		Time	On claim	
		Age		
		Gender		
		Other		
**Denominator**	Patients with headache and no indication of epilepsy or convulsions within past 1 year and no other headache diagnosis within the past 2 years	ICD-10	G43, G44, R51	**Epilepsy/convulsions:** G40, R25, R56<br>**Other headache diagnosis:** G43, G44, R51
		BETOS		
		CPT/HCPCS		
		MS-DRGs/Drug		
		Time	On claim	**Epilepsy/convulsions:** Within the past 1 year<br>**Other headache diagnosis:** Within the past 2 years
		Age		
		Gender		
		Other		

Definition – Sensitivity Analysis		Codes	Include	Exclude
**Numerator**	Patients that received an electroencephalogram (EEG)	ICD-10		
		BETOS		

© 2025 Kim DD et al. *JAMA Health Forum*.

		CPT/HCPCS	95812, 95813, 95816, 95819, 95822, 95827, 95830, 95957	
		MS-DRGs/Drug		
		Time	On claim	
		Age		
		Gender		
		Other		
**Denominator**	Patients with headache and no indication of epilepsy or convulsions within the past 1 year	ICD-10	G43, G44, R51	G40, R25, R56
		BETOS		
		CPT/HCPCS		
		MS-DRGs/Drug		
		Time	On claim	Within the past 1 year
		Age		
		Gender		
		Other		

© 2025 Kim DD et al. *JAMA Health Forum*.

26. Electromyography for low back pain (LBP)

Recommendation	Clinical Source(s)	Low-Value Study Source(s)
Do not perform electromyography for low back pain		Fleming 2022[23]

Definition – Base Case		Codes	Include	Exclude
**Numerator**	Patients that received electromyography	ICD-10		
		BETOS		
		CPT/HCPCS	95860, 95861, 95863, 95864, 95869, 95885, 95886	
		MS-DRGs/Drug		
		Time	On claim	
		Age		
		Gender		
		Other		
**Denominator**	Patients with low back pain and without leg pain or sciatica in past 12 months	ICD-10	M43.27, M43.28, M46.47, M47.819, M51.27, M51.36, M51.87, M99.83, M51.26, M51.37, M51.86, M53.2X7, M53.2X8, M53.3, M54.5, M54.89, M54.9, M99.03, M99.04, M99.84, S335XXA, S336XXA, S338XXA, S339XXA	G83.4, M54.30, M47.2x, M54.3x, M50.1xx, M54.4x, M51.1x, M79.2, M54.14, M79.604, M54.15, M79.605, M54.16, M79.606, M54.17, M54.30
		BETOS		
		CPT/HCPCS		
		MS-DRGs/Drug		
		Time	Within past 1 year	Within past 1 year
		Age		
		Gender		
		Other		

Definition – Sensitivity Analysis		Codes	Include	Exclude
**Numerator**	Patients that received electromyography	ICD-10	95860, 95861, 95863, 95864, 95869, 95885, 95886	

© 2025 Kim DD et al. *JAMA Health Forum*.

		BETOS		
		CPT/HCPCS		
		MS-DRGs/Drug		
		Time	On claim	
		Age		
		Gender		
		Other		
**Denominator**	Patients with low back pain and without leg pain or sciatica in past 12 months	ICD-10	M43.27, M43.28, M46.46, M46.47, M47.20, M47.26, M47.27, M47.28, M47.816, M47.817, M47.818, M47.819, M47.896, M47.897, M47.898, M47.899, M47.9, M51.16, M51.17, M51.26, M51.27, M51.34, M51.35, M51.36, M51.37, M51.46, M51.47, M51.86, M51.87, M53.2X7, M53.2X8, M53.3, M53.86, M53.87, M53.88, M54.30, M54.31, M54.32, M54.40, M54.41, M54.42, M54.5, M54.89, M54.9, M99.03, M99.04, M99.83, M99.84, S33.5XXA, S33.6XXA, S33.8XXA, S33.9XXA	G83.4, M54.30, M47.2x, M54.3x, M50.1xx, M54.4x, M51.1x, M79.2, M54.14, M79.604, M54.15, M79.605, M54.16, M79.606, M54.17, M54.30
		BETOS		
		CPT/HCPCS		
		MS-DRGs/Drug		
		Time	Within past 1 year	Within past 1 year
		Age		
		Gender		
		Other		

© 2025 Kim DD et al. *JAMA Health Forum*.

27. Head imaging for headache

Recommendation	Clinical Source(s)	Low-Value Study Source(s)
Do not perform brain CT or MRI imaging for non-post-traumatic, non-thunderclap headache	CW American College of Radiology (2017/2012)	Park (2021)[2], Schwartz (2018)[5], Barnett (2017)[22], Carter (2017)[6], Reid (2017)[14], Charlesworth (2016)[15]

Definition – Base Case		Codes	Include	Exclude
**Numerator**	Patients that received a CT or MRI of head or brain	ICD-10		
		BETOS		
		CPT/HCPCS	70450, 70460, 70470, 70551-70553	
		MS-DRGs/Drug		
		Time	On claim	
		Age		
		Gender		
		Other		
**Denominator**	Patients with headache and no other warranted diagnosis<br><br>Warranted diagnosis: post-traumatic or thunderclap headache, cancer, migraine with hemiplegia or infarction, giant cell arteritis, epilepsy or convulsions, cerebrovascular diseases including stroke/TIA and subarachnoid hemorrhage, head or face trauma, altered mental status, nervous and musculoskeletal system symptoms including gait abnormality, meningismus, disturbed skin sensation and speech deficits, personal history of stroke/TIA or cancer	ICD-10	G43.0, G43.1, G43.5, G43.7, G43.8, G43.9, G43.A, G43.B, G43.C, G43.D, G44.0, G44.1, G44.2, G44.4, G44.51, G44.52, G44.59, G44.8, R51	C00-C99, D00-D09, D37-49, G40, G43.4, G43.6, G44.3, G44.53, G45, G46, I60-I69, L08.89, M31.5, M31.6, Q85, R20, R25-R29, R41.0, R41.4, R41.82, R41.842, R43, R47, R56, R68.3, S00-S02, S05, S06, S08-S10, S16, S19, Z85, Z86.73
		BETOS		
		CPT/HCPCS		
		MS-DRGs/Drug		
		Time	On claim	On claim
		Age		
		Gender		
		Other		

© 2025 Kim DD et al. *JAMA Health Forum*.

No definition for sensitivity analysis

© 2025 Kim DD et al. *JAMA Health Forum*.

28. Head imaging for syncope

Recommendation	Clinical Source(s)	Low-Value Study Source(s)
Do not perform CT or MRI imaging for a diagnosis of syncope	CW American College of Physicians (2012)	Chalmers (2021)[21], Ganguli (2021)[1], Schwartz (2018)[5], Carter (2017)[6], Reid (2017)[14], Charlesworth (2016)[15]

Definition – Base Case		Codes	Include	Exclude
**Numerator**	Patients that received a CT or MRI of head or brain	ICD-10		
		BETOS		
		CPT/HCPCS	70450, 70460, 70470, 70551-70553	
		MS-DRGs/Drug		
		Time	On claim	
		Age		
		Gender		
		Other		
**Denominator**	Patients with syncope and without a warranted diagnosis<br><br>Warranted diagnosis: epilepsy or convulsions, cerebrovascular diseases including stroke/TIA and subarachnoid hemorrhage, head or face trauma, altered mental status, nervous and musculoskeletal system symptoms including gait abnormality, meningismus, disturbed skin sensation and speech deficits, personal history of stroke/TIA	ICD-10	R55, T67.1XXA	G40, G45, G46, I60-I69, L08.89, R20, R25-R29, R41.0, R41.4, R41.82, R43, R47, R56, R68.3, S00-S02, S05, S06, S08-S10, S16, S19, Z85, Z86.73
		BETOS		
		CPT/HCPCS		
		MS-DRGs/Drug		
		Time	On claim	On claim
		Age		
		Gender		
		Other		

No definition for sensitivity analysis

© 2025 Kim DD et al. *JAMA Health Forum*.

29. Imaging for benign prostatic hyperplasia (BPH)

Recommendation	Clinical Source(s)	Low-Value Study Source(s)
Do not perform upper tract imaging in patients with benign prostatic hyperplasia (BPH)	CW American Urological Association (2017/2012)	Colla (2015)[10]

Definition – Base Case		Codes	Include	Exclude
**Numerator**	Patients that receive upper-tract imaging: intravenous pyelogram, CT scan abdomen, MRI abdomen, diagnostic ultrasound of abdomen	ICD-10		
		BETOS		
		CPT/HCPCS	74400, 74405, 74410-74425, 74150-74170, 74181-74183, 76700, 76705, 76770, 76775	
		MS-DRGs/Drug		
		Time	On claim	
		Age		
		Gender		
		Other		
**Denominator**	Patients with benign prostatic hyperplasia (BPH) and without warranted diagnosis<br><br>Warranted diagnosis: chronic renal failure, nephritis, nephrotic syndrome, and nephrosis, other pyelonephritis or pyonephrosis not specified as acute or chronic, calculus of kidney and ureter, kidney stones, urinary tract infections, hematuria, fever, urinary retention, abdominal pain, cancer except non-melanoma skin cancer	ICD-10	N39.41, N40.0, N40.1, R32, R33.9, R35.0, R35.1, R39.12, R39.14	B52.0, E08.2, E09.2, I12.0, M32.14, M32.15, M35.04, N00-N08, N11.9, N12, N13.2, N13.6, N13.9, N14, N15.0, N15.8, N15.9, N16-N20, N22, N25, N26.1, N26.9, N27, N39.0, R10.0, R10.1, R10.2, R10.3, R10.84, R10.9, R31, R33.0, R33.8, R39.14, R50, R68.0, R68.83
		BETOS		
		CPT/HCPCS		
		MS-DRGs/Drug		
		Time	On claim	On claim
		Age		
		Gender		
		Other		HCC 7-10

No definition for sensitivity analysis

© 2025 Kim DD et al. *JAMA Health Forum*.

30. Imaging for plantar fasciitis

Recommendation	Clinical Source(s)	Low-Value Study Source(s)
Do not perform radiographic or MR imaging with diagnosis of plantar fasciitis occurring within 2 weeks of initial foot pain diagnosis		Schwartz (2018)[5], Reid (2017)[14], Charlesworth (2016)[15]

Definition – Base Case		Codes	Include	Exclude
**Numerator**	Patients that received foot radiograph, foot MRI, or extremity ultrasound	ICD-10		
		BETOS		
		CPT/HCPCS	73620, 73630, 73650, 73718, 73719, 73720, 76880, 76881, 76882	
		MS-DRGs/Drug		
		Time	On claim	
		Age		
		Gender		
		Other		
**Denominator**	Patients with reported foot pain **and** with plantar fasciitis diagnosis within two weeks of initial foot pain	ICD-10	**Plantar fasciitis:** M72.2, M72.9<br>**Foot pain:** M25.57, M79.67	
		BETOS		
		CPT/HCPCS		
		MS-DRGs/Drug		
		Time	**Plantar fasciitis:** On claim<br>**Foot pain:** Within past 2 weeks	
		Age		
		Gender		
		Other		

Definition – Sensitivity Analysis		Codes	Include	Exclude
**Numerator**	Patients that received foot radiograph, foot MRI, or extremity ultrasound	ICD-10		
		BETOS		
		CPT/HCPCS	73620, 73630, 73650, 73718, 73719, 73720, 76880, 76881, 76882	
		MS-DRGs/Drug		

© 2025 Kim DD et al. *JAMA Health Forum*.

		Time	On claim	
		Age		
		Gender		
		Other		
**Denominator**	Patients with reported foot pain and with plantar fasciitis diagnosis within four weeks of initial foot pain	ICD-10	**Plantar fasciitis:** M72.2, M72.9<br>**Foot pain:** M25.57, M79.67	
		BETOS		
		CPT/HCPCS		
		MS-DRGs/Drug		
		Time	**Plantar fasciitis:** On claim<br>**Foot pain:** Within past 4 weeks	
		Age		
		Gender		
		Other		

© 2025 Kim DD et al. *JAMA Health Forum*.

31. Low back pain (LBP) imaging

Recommendation	Clinical Source(s)	Low-Value Study Source(s)
Don't do imaging for low back pain within the first six weeks without another diagnosis that warrants imaging	CW Canadian Association of Radiologists (2021), CW College of Family Physicians of Canada (2020), CW North American Spine Society (2019/2013), CW American Academy of Family Physicians (2012)	Ganguli (2021)[1], Sanghavi (2021)[3], Kool (2020)[11], Koehlmoos (2019)[18], Oakes (2019)[19], Rosenthal (2018)[16], Schwartz (2018)[5], VBID (2018)[12], Carter (2017)[6], Barnett (2017)[22], Pendrith (2017)[8], Reid (2017)[14], Schpero (2017)[9], Charlesworth (2016)[15], Colla (2015)[10], Segal (2014)[20]

Definition – Base Case		Codes	Include	Exclude
**Numerator**	Patients that received imaging of the lower back	ICD-10		
		BETOS		
		CPT/HCPCS	72010, 72020, 72052, 72100, 72110, 72114, 72120, 72200, 72202, 72220, 72131, 72132, 72133, 72141, 72142, 72146, 72147, 72148, 72149, 72156, 72157, 72158	
		MS-DRGs/Drug		
		Time	On claim	
		Age		
		Gender		
		Other		
**Denominator**	Patients with lower back pain and without a warranted diagnosis<br><br>Warranted diagnosis: cancer, external injury, trauma, IV drug abuse, neurologic impairment, osteomyelitis, myelopathy, neuritis, radiculopathy, intraspinal abscess, fever, weight loss, malaise, night sweats, anemia not due to	ICD-10	M43.27, M43.28, M46.46, M46.47, M47.20, M47.26, M47.27, M47.28, M47.816, M47.817, M47.818, M47.819, M47.896, M47.897, M47.898, M47.899, M47.9, M51.16, M51.17, M51.26, M51.27, M51.34, M51.35, M51.36, M51.37, M51.46, M51.47, M51.86, M51.87, M53.2X7, M53.2X8, M53.3, M53.86, M53.87, M53.88, M54.30, M54.31, M54.32, M54.40, M54.41, M54.42, M54.5, M54.89, M54.9, M99.03, M99.04, M99.83, M99.84,	A15, A17, A18, A19, A40, A41, A42.7, C00-C96, D00-D09, D37-D49, D64.9, F11, F13-F15, G06, G07, G83.4, G93.3, I33, I39, L59.9, M46, M47.10, M47.16, M48, M51.0, M51.9, M54.1, M67.90, M79.2, M80, M84, M86, M89.6, M90.8, M97, M99.1, Q85.0, R50, R53, R61, R63, R68.0, R86.3, S00-S99, T07, T14-T28, T33-T85, T88, V00-V99, W00-W99, X00-X99, Y00-Y69, Y83, Y84, Y92, Y99

© 2025 Kim DD et al. *JAMA Health Forum*.

	blood loss, tuberculosis, septicemia, endocarditis		S33.5XXA, S33.6XXA, S33.8XXA, S33.9XXA	
		BETOS		
		CPT/HCPCS		
		MS-DRGs/Drug		
		Time	Within the past 6 weeks	Within the past 6 weeks
		Age		
		Gender		
		Other		

**Definition – Sensitivity Analysis**		**Codes**	**Include**	**Exclude**
**Numerator**	Patients that received imaging of the lower back	ICD-10		
		BETOS		
		CPT/HCPCS	72010, 72020, 72052, 72100, 72110, 72114, 72120, 72200, 72202, 72220, 72131, 72132, 72133, 72141, 72142, 72146, 72147, 72148, 72149, 72156, 72157, 72158	
		MS-DRGs/Drug		
		Time	On claim	
		Age		
		Gender		
		Other		
**Denominator**	Patients with lower back pain and without a warranted diagnosis<br><br>Warranted diagnosis: cancer, external injury, trauma, IV drug abuse, neurologic impairment, osteomyelitis, myelopathy, neuritis, radiculopathy, intraspinal abscess	ICD-10	M43.27, M43.28, M46.46, M46.47, M47.20, M47.26, M47.27, M47.28, M47.816, M47.817, M47.818, M47.819, M47.896, M47.897, M47.898, M47.899, M47.9, M51.16, M51.17, M51.26, M51.27, M51.34, M51.35, M51.36, M51.37, M51.46, M51.47, M51.86, M51.87, M53.2X7, M53.2X8, M53.3, M53.86, M53.87, M53.88, M54.30, M54.31, M54.32, M54.40, M54.41, M54.42, M54.5, M54.89, M54.9, M99.03, M99.04, M99.83, M99.84,	C00-C96, D00-D09, D37-D49, F11, F13-F15, G06, G07, G83.4, G93.3, L59.9, M46, M47.10, M47.16, M48, M51.0, M51.9, M54.1, M67.90, M79.2, M80, M84, M86, M89.6, M90.8, M97, M99.1, Q85.0, S00-S99, T07, T14-T28, T33-T85, T88, V00-V99, W00-W99, X00-X99, Y00-Y69, Y83, Y84, Y92, Y99

© 2025 Kim DD et al. *JAMA Health Forum*.

			S33.5XXA, S33.6XXA, S33.8XXA, S33.9XXA	
		BETOS		
		CPT/HCPCS		
		MS-DRGs/Drug		
		Time	Within the past 6 weeks	Within the past 6 weeks
		Age		
		Gender		
		Other		

© 2025 Kim DD et al. *JAMA Health Forum*.

32. MRI for rheumatoid arthritis

Recommendation	Clinical Source(s)	Low-Value Study Source(s)
Do not perform MRI for rheumatoid arthritis		Fleming (2022)[23]

Definition – Base Case		Codes	Include	Exclude
**Numerator**	Patients that received MRI of peripheral joint	ICD-10		
		BETOS		
		CPT/HCPCS	73221, 73222, 73223, 73721, 73722, 73723	
		MS-DRGs/Drug		
		Time		
		Age		
		Gender		
		Other		
**Denominator**	Patients with rheumatoid arthritis	ICD-10	M05.411, M05.551, M05.811, M06.042, M06.272, M06.831, M05.412, M05.552, M05.812, M06.049, M06.279, M06.832, M05.419, M05.559, M05.819, M06.051, M06.28, M06.839, M05.421, M05.561, M05.821, M06.052, M06.29, M06.841, M05.422, M05.562, M05.822, M06.059, M06.30, M06.842, M05.429, M05.569, M05.829, M06.061, M06.311, M06.849, M05.431, M05.571, M05.831, M06.062, M06.312, M06.851, M05.432, M05.572, M05.832, M06.069, M06.319, M06.852, M05.439, M05.579, M05.839, M06.071, M06.321, M06.859, M05.441, M05.59, M05.841, M06.072, M06.322, M06.861, M05.442, M05.70, M05.842, M06.079, M06.329, M06.862, M05.449, M05.711, M05.849, M06.08, M06.331, M06.869, M05.451, M05.712, M05.851, M06.09, M06.332, M06.871, M05.452, M05.719,	

© 2025 Kim DD et al. *JAMA Health Forum*.

			M05.852, M06.0A, M06.339, M06.872, M05.459, M05.721, M05.859, M06.0A, M06.341, M06.879, M05.461, M05.722, M05.861, M06.20, M06.342, M06.88, M05.462, M05.729, M05.862, M06.211, M06.349, M06.89, M05.469, M05.731, M05.869, M06.212, M06.351, M06.8A, M05.471, M05.732, M05.871, M06.219, M06.352, M06.8A, M05.472, M05.739, M05.872, M06.221, M06.359, M06.9, M05.479, M05.741, M05.879, M06.222, M06.361, M05.49, M05.742, M05.89, M06.229, M06.362, M05.50, M05.749, M05.8A, M06.231, M06.369, M05.511, M05.751, M05.9, M06.232, M06.371, M05.512, M05.752, M06.00, M06.239, M06.372, M05.519, M05.759, M06.011, M06.241, M06.379, M05.521, M05.761, M06.012, M06.242, M06.38, M05.522, M05.762, M06.019, M06.249, M06.39, M05.529, M05.769, M06.021, M06.251, M06.80, M05.531, M05.771, M06.022, M06.252, M06.811, M05.532, M05.772, M06.029, M06.259, M06.812, M05.539, M05.779, M06.031, M06.261, M06.819, M05.541, M05.79, M06.032, M06.262, M06.821, M05.542, M05.7A, M06.039, M06.269, M06.822, M05.549, M05.80, M06.041, M06.271, M06.829	
		BETOS		
		CPT/HCPCS		
		MS-DRGs/Drug		
		Time	On claim	
		Age		
		Gender		

© 2025 Kim DD et al. *JAMA Health Forum*.

		Other		

No definition for sensitivity analysis

© 2025 Kim DD et al. *JAMA Health Forum*.

33. Advanced imaging for cardiac screening

Recommendation	Clinical Source(s)	Low-Value Study Source(s)
Do not perform advanced imaging (CT, MRI, PET) on low-risk, asymptomatic patients	CW American College of Cardiology (2021), CW Society of Cardiovascular Computed Tomography (2021/2013), CW American Society of Echocardiography (2013), CW American Academy of Family Physicians (2012), CW American Society of Nuclear Cardiology (2012)	Colla (2015)[10], Schpero (2017)[9]

Definition – Base Case		Codes	Include	Exclude
**Numerator**	Patients that received advanced imaging (CT, MRI, PET)	ICD-10		
		BETOS		
		CPT/HCPCS	0144T, 0145T, 0146T, 0147T, 0148T, 0149T, 0150T, 75552, 75553, 75554, 75555, 75556, 75557, 75558, 75559, 75561, 75562, 75565, 75571, 75572, 75573, 75574, 78451, 78452, 78453, 78454, 78460, 78461, 78464, 78465, 78478, 78480, 78459, 78481, 78483, 78491, 78492, 78494, 78496, 78499	
		MS-DRGs/Drug		
		Time	On claim	
		Age		
		Gender		
		Other		
**Denominator**	Patients that have no high-risk indications or symptomatic indications<br><br>**High risk indications:** HIV/AIDS, cancer, diabetes, drug/alcohol dependence, cardio-respiratory failure and shock, congestive heart failure, acute myocardial infarction, unstable angina and	ICD-10		**Symptomatic indications:** C00-C97, D00-D48, G00-G13, G35-G37, G40, G41, G45, G46, G47, G90-G99, I05-I15, I20-I52, I70-I79, I95-I99, J40-J47, J60-J70, J80-J86, J90-J99, N10-N19, R10, R53, T36-T65, X40-X57, Y10-Y19
		BETOS		
		CPT/HCPCS		
		MS-DRGs/Drug		
		Time		**High risk indications:** All past data

© 2025 Kim DD et al. *JAMA Health Forum*.

	other acute ischemic heart disease, angina pectoris/old myocardial infarction, specified heart arrhythmias, ischemic or unspecified stroke, hemiplegia/hemiparesis, vascular disease with complications, vascular disease, chronic obstructive pulmonary disease, renal failure<br>**Symptomatic indications:** proximal fractures, pulmonary and chest conditions, esophageal conditions except mild and chronic, cardiac conditions, end stage renal disease and dialysis, infections including sepsis but not urinary tract infections or pneumonia, anemia and bleeding conditions except microscopic hematuria, abdominal conditions or pain but not benign tumor of the colon, psychiatric conditions, acute central nervous system conditions and seizure or epilepsy conditions but not chronic conditions or migraine, drug toxicity and poisoning, transplants, malignant hypertension, cancer, conditions involving arterial and pulmonary emboli but not peripheral deep vein thrombosis, falls, autoimmune conditions, pain in thorax or shoulder but not specific shoulder conditions			**Symptomatic indications:** On claim
		Age		
		Gender		
		Other		**High risk indications:** HCC: 1, 7-10, 15-19, 52, 79, 80, 81, 82, 83, 92, 96, 100, 104, 105, 108, 113

© 2025 Kim DD et al. *JAMA Health Forum*.

Definition – Sensitivity Analysis		Codes	Include	Exclude
**Numerator**	Patients that received advanced imaging (CT, MRI, PET)	ICD-10		
		BETOS		
		CPT/HCPCS	0144T, 0145T, 0146T, 0147T, 0148T, 0149T, 0150T, 75552, 75553, 75554, 75555, 75556, 75557, 75558, 75559, 75561, 75562, 75565, 75571, 75572, 75573, 75574, 78451, 78452, 78453, 78454, 78460, 78461, 78464, 78465, 78478, 78480, 78459, 78481, 78483, 78491, 78492, 78494, 78496, 78499	
		MS-DRGs/Drug		
		Time	On claim	
		Age		
		Gender		
		Other		
**Denominator**	Patients that have no high-risk indications<br><br>**High risk indications:** See 33. Advanced imaging for cardiac screening base case definition	ICD-10		
		BETOS		
		CPT/HCPCS		
		MS-DRGs/Drug		
		Time		All past data
		Age		
		Gender		
		Other		**High risk indications:** HCC: 1, 7-10, 15-19, 52, 79, 80, 81, 82, 83, 92, 96, 100, 104, 105, 108, 113

© 2025 Kim DD et al. *JAMA Health Forum*.

34. Cardiovascular stress test for cardiac screening

Recommendation	Clinical Source(s)	Low-Value Study Source(s)
Do not perform cardiovascular stress test on low-risk, asymptomatic patients	CW American College of Cardiology (2021), CW Society of Cardiovascular Computed Tomography (2021/2013), USPSTF Jonas (2018), CW American Society of Echocardiography (2013), CW American Academy of Family Physicians (2012), CW American Society of Nuclear Cardiology (2012), USPSTF Moyer (2012)	Ganguli (2021)[1], Rosenthal (2018)[16], Schpero (2017)[9], Colla (2015)[10]

Definition – Base Case		Codes	Include	Exclude
**Numerator**	Patients that received a cardiovascular stress test	ICD-10		
		BETOS		
		CPT/HCPCS	75560, 75563, 75564, 93015, 93016, 93017, 93018, 93024, 93350, 93351, 93352	
		MS-DRGs/Drug		
		Time	On claim	
		Age		
		Gender		
		Other		
**Denominator**	Patients that have no high-risk indications or symptomatic indications<br><br>**High risk indications:** See 33. Advanced imaging for cardiac screening base case definition<br>**Symptomatic indications:** See 33. Advanced imaging for cardiac screening base case definition	ICD-10		**Symptomatic indications:** C00-C97, D00-D48, G00-G13, G35-G37, G40, G41, G45, G46, G47, G90-G99, I05-I15, I20-I52, I70-I79, I95-I99, J40-J47, J60-J70, J80-J86, J90-J99, N10-N19, R10, R53, T36-T65, X40-X57, Y10-Y19
		BETOS		
		CPT/HCPCS		
		MS-DRGs/Drug		
		Time		**High risk indications:** All past data<br>**Symptomatic indications:** On claim
		Age		
		Gender		

© 2025 Kim DD et al. *JAMA Health Forum*.

		Other		**High risk indications:** HCC: 1, 7-10, 15-19, 52, 79, 80, 81, 82, 83, 92, 96, 100, 104, 105, 108, 113

**Definition – Sensitivity Analysis**		**Codes**	**Include**	**Exclude**
**Numerator**	Patients that received a cardiovascular stress test	ICD-10		
		BETOS		
		CPT/HCPCS	75560, 75563, 75564, 93015, 93016, 93017, 93018, 93024, 93350, 93351, 93352	
		MS-DRGs/Drug		
		Time	On claim	
		Age		
		Gender		
		Other		
**Denominator**	Patients that have no high-risk indications<br><br>**High risk indications:** See 33. Advanced imaging for cardiac screening base case definition	ICD-10		
		BETOS		
		CPT/HCPCS		
		MS-DRGs/Drug		
		Time		All past data
		Age		
		Gender		
		Other		**High risk indications:** HCC: 1, 7-10, 15-19, 52, 79, 80, 81, 82, 83, 92, 96, 100, 104, 105, 108, 113

© 2025 Kim DD et al. *JAMA Health Forum*.

35. Echocardiogram for cardiac screening

Recommendation	Clinical Source(s)	Low-Value Study Source(s)
Do not perform echocardiogram on low-risk, asymptomatic patients	CW American College of Cardiology (2021), CW Society of Cardiovascular Computed Tomography (2021/2013), CW American Society of Echocardiography (2013), CW American Academy of Family Physicians (2012), CW American Society of Nuclear Cardiology (2012)	Rosenthal (2018)[16], Schpero (2017)[9], Colla (2015)[10]

Definition – Base Case		Codes	Include	Exclude
**Numerator**	Patients that received an echocardiogram	ICD-10		
		BETOS		
		CPT/HCPCS	93303, 93304, 93306, 93307, 93308, 93320, 93321, 93325	
		MS-DRGs/Drug		
		Time	On claim	
		Age		
		Gender		
		Other		
**Denominator**	Patients that have no high-risk indications or symptomatic indications<br><br>**High risk indications:** See 33. Advanced imaging for cardiac screening base case definition<br>**Symptomatic indications:** See 33. Advanced imaging for cardiac screening base case definition	ICD-10		**Symptomatic indications:** C00-C97, D00-D48, G00-G13, G35-G37, G40, G41, G45, G46, G47, G90-G99, I05-I15, I20-I52, I70-I79, I95-I99, J40-J47, J60-J70, J80-J86, J90-J99, N10-N19, R10, R53, T36-T65, X40-X57, Y10-Y19
		BETOS		
		CPT/HCPCS		
		MS-DRGs/Drug		
		Time		**High risk indications:** All past data<br>**Symptomatic indications:** On claim
		Age		
		Gender		
		Other		**High risk indications:** HCC: 1, 7-10, 15-19, 52, 79, 80, 81, 82, 83, 92, 96, 100, 104, 105, 108, 113

© 2025 Kim DD et al. *JAMA Health Forum*.

Definition – Sensitivity Analysis		Codes	Include	Exclude
**Numerator**	Patients that received an echocardiogram	ICD-10		
		BETOS		
		CPT/HCPCS	93303, 93304, 93306, 93307, 93308, 93320, 93321, 93325	
		MS-DRGs/Drug		
		Time	On claim	
		Age		
		Gender		
		Other		
**Denominator**	Patients that have no high-risk indications<br><br>**High risk indications:** See 33. Advanced imaging for cardiac screening base case definition	ICD-10		
		BETOS		
		CPT/HCPCS		
		MS-DRGs/Drug		
		Time		All past data
		Age		
		Gender		
		Other		**High risk indications:** HCC: 1, 7-10, 15-19, 52, 79, 80, 81, 82, 83, 92, 96, 100, 104, 105, 108, 113

© 2025 Kim DD et al. *JAMA Health Forum*.

36. Electrocardiogram for cardiac screening

Recommendation	Clinical Source(s)	Low-Value Study Source(s)
Do not perform electrocardiogram on low-risk, asymptomatic patients	CW American College of Cardiology (2021), CW Society of Cardiovascular Computed Tomography (2021/2013), USPSTF Jonas (2018), CW American Society of Echocardiography (2013), CW American Academy of Family Physicians (2012), CW American Society of Nuclear Cardiology (2012), USPSTF Moyer (2012)	Ganguli (2021)[1], Bouck (2018)[4], Rosenthal (2018)[16], Mafi (2017)[17], Schpero (2017)[9], Colla (2015)[10]

Definition – Base Case		Codes	Include	Exclude
**Numerator**	Patients that received an electrocardiogram	ICD-10		
		BETOS		
		CPT/HCPCS	3120F, 93000, 93005, 93010, G0366, G0367, G0368, G0403, G0404, G0405	
		MS-DRGs/Drug		
		Time	On claim	
		Age		
		Gender		
		Other		
**Denominator**	Patients that have no high-risk indications or symptomatic indications<br><br>**High risk indications:** See 33. Advanced imaging for cardiac screening base case definition<br>**Symptomatic indications:** See 33. Advanced imaging for cardiac screening base case definition	ICD-10		**Symptomatic indications:** C00-C97, D00-D48, G00-G13, G35-G37, G40, G41, G45, G46, G47, G90-G99, I05-I15, I20-I52, I70-I79, I95-I99, J40-J47, J60-J70, J80-J86, J90-J99, N10-N19, R10, R53, T36-T65, X40-X57, Y10-Y19
		BETOS		
		CPT/HCPCS		
		MS-DRGs/Drug		
		Time		**High risk indications:** All past data<br>**Symptomatic indications:** On claim
		Age		
		Gender		

© 2025 Kim DD et al. *JAMA Health Forum*.

		Other		**High risk indications:** HCC: 1, 7-10, 15-19, 52, 79, 80, 81, 82, 83, 92, 96, 100, 104, 105, 108, 113

**Definition – Sensitivity Analysis**		**Codes**	**Include**	**Exclude**
**Numerator**	Patients that received an electrocardiogram	ICD-10		
		BETOS		
		CPT/HCPCS	3120F, 93000, 93005, 93010, G0366, G0367, G0368, G0403, G0404, G0405	
		MS-DRGs/Drug		
		Time	On claim	
		Age		
		Gender		
		Other		
**Denominator**	Patients that have no high-risk indications<br><br>**High risk indications:** See 33. Advanced imaging for cardiac screening base case definition	ICD-10		
		BETOS		
		CPT/HCPCS		
		MS-DRGs/Drug		
		Time		All past data
		Age		
		Gender		
		Other		**High risk indications:** HCC: 1, 7-10, 15-19, 52, 79, 80, 81, 82, 83, 92, 96, 100, 104, 105, 108, 113

© 2025 Kim DD et al. *JAMA Health Forum*.

37. Inferior vena cava (IVC) filter

Recommendation	Clinical Source(s)	Low-Value Study Source(s)
Do not perform IVC filter placement	CW Society for Vascular Surgery (2015)	Chalmers (2021)[21], Badgery-Parker (2018)[24], Schwartz (2018)[5], Reid (2017)[14]

Definition – Base Case		Codes	Include	Exclude
**Numerator**	Patients that received an inferior vena cava (IVC) placement	ICD-10		
		BETOS		
		CPT/HCPCS	37191, 37192, 75940	
		MS-DRGs/Drug		
		Time	On claim	
		Age		
		Gender		
		Other		
**Denominator**	Patients without a history of or current pulmonary embolism or deep vein thrombosis in previous year	ICD-10		I26, Z86.711, I82.49, I82.59,
		BETOS		
		CPT/HCPCS		
		MS-DRGs/Drug		
		Time		Within the past 1 year
		Age		
		Gender		
		Other		

Definition – Sensitivity Analysis		Codes	Include	Exclude
**Numerator**	Patients that received an inferior vena cava (IVC) placement	ICD-10		
		BETOS		
		CPT/HCPCS	37191, 37192, 75940	
		MS-DRGs/Drug		
		Time	On claim	
		Age		
		Gender		
		Other		
**Denominator**	All patients	ICD-10		

© 2025 Kim DD et al. *JAMA Health Forum*.

		BETOS		
		CPT/HCPCS		
		MS-DRGs/Drug		
		Time		
		Age		
		Gender		
		Other		

© 2025 Kim DD et al. *JAMA Health Forum*.

38. Percutaneous coronary intervention (PCI)

Recommendation	Clinical Source(s)	Low-Value Study Source(s)
Do not perform percutaneous coronary intervention (PCI) with balloon angioplasty or stent placement, not associated with an ER visit, for patients with stable coronary disease	CW Society for Cardiovascular Angiography and Interventions (2018/2014)	Chalmers (2021)[21], Ganguli (2021)[1], Badgery-Parker (2018)[24], Schwartz (2018)[5]

Definition – Base Case		Codes	Include	Exclude
**Numerator**	Patients that received percutaneous coronary intervention (PCI) with balloon angioplasty or stent placement	ICD-10	ICD-10-PCS: 0270446, 027044Z, 0270456, 027045Z, 0270466, 027046Z, 0270476, 027047Z, 02704D6, 02704DZ, 02704E6, 02704EZ, 02704F6, 02704FZ, 02704G6, 02704GZ, 02704T6, 02704TZ, 02704Z6, 02704ZZ, 0271446, 027144Z, 0271456, 027145Z, 0271466, 027146Z, 0271476, 027147Z, 02714D6, 02714DZ, 02714E6, 02714EZ, 02714F6, 02714FZ, 02714G6, 02714GZ, 02714T6, 02714TZ, 02714Z6, 02714ZZ, 0272446, 027244Z, 0272456, 027245Z, 0272466, 027246Z, 0272476, 027247Z, 02724D6, 02724DZ, 02724E6, 02724EZ, 02724F6, 02724FZ, 02724G6, 02724GZ, 02724T6, 02724TZ, 02724Z6, 02724ZZ, 0273446, 027344Z, 0273456, 027345Z, 0273466, 027346Z, 0273476, 027347Z, 02734D6, 02734DZ, 02734E6, 02734EZ, 02734F6, 02734FZ, 02734G6, 02734GZ, 02734T6, 02734TZ, 02734Z6, 02734ZZ	
		BETOS		
		CPT/HCPCS	92928, 92929, 92933, 92934, 92937, 92938, 92941, 92943, 92944	**Emergency care**: 99281-99285, 99288
		MS-DRGs/Drug		
		Time	On claim	On claim
		Age		

© 2025 Kim DD et al. *JAMA Health Forum*.

		Gender		
		Other		
**Denominator**	Patients with stable coronary disease (defined as ischemic heart disease or acute myocardial infarction more than 6 months before PCI) and without unstable angina or myocardial infarction in two weeks before claim	ICD-10	I24.8, I24.9, I25, I21, I22, I23	I20.0, I21, I22, I23, I25.110, I25.700, I25.710
		BETOS		
		CPT/HCPCS		
		MS-DRGs/Drug		
		Time	At least 6 months before claim	Within the past 2 weeks
		Age		
		Gender		
		Other		

**Definition – Sensitivity Analysis**		**Codes**	**Include**	**Exclude**
**Numerator**	Patients that received percutaneous coronary intervention (PCI) with balloon angioplasty or stent placement	ICD-10	ICD-10-PCS: 0270446, 027044Z, 0270456, 027045Z, 0270466, 027046Z, 0270476, 027047Z, 02704D6, 02704DZ, 02704E6, 02704EZ, 02704F6, 02704FZ, 02704G6, 02704GZ, 02704T6, 02704TZ, 02704Z6, 02704ZZ, 0271446, 027144Z, 0271456, 027145Z, 0271466, 027146Z, 0271476, 027147Z, 02714D6, 02714DZ, 02714E6, 02714EZ, 02714F6, 02714FZ, 02714G6, 02714GZ, 02714T6, 02714TZ, 02714Z6, 02714ZZ, 0272446, 027244Z, 0272456, 027245Z, 0272466, 027246Z, 0272476, 027247Z, 02724D6, 02724DZ, 02724E6, 02724EZ, 02724F6, 02724FZ, 02724G6, 02724GZ, 02724T6, 02724TZ, 02724Z6, 02724ZZ, 0273446, 027344Z, 0273456, 027345Z, 0273466, 027346Z, 0273476, 027347Z, 02734D6, 02734DZ, 02734E6, 02734EZ, 02734F6, 02734FZ, 02734G6, 02734GZ, 02734T6, 02734TZ, 02734Z6, 02734ZZ	
		BETOS		

© 2025 Kim DD et al. *JAMA Health Forum*.

		CPT/HCPCS	92928, 92929, 92933, 92934, 92937, 92938, 92941, 92943, 92944	**Emergency care**: 99281-99285, 99288
		MS-DRGs/Drug		
		Time	On claim	On claim
		Age		
		Gender		
		Other		
**Denominator**	Patients with stable coronary disease (defined as ischemic heart disease or acute myocardial infarction more than 6 months before PCI)	ICD-10	I24.8, I24.9, I25, I21, I22, I23	
		BETOS		
		CPT/HCPCS		
		MS-DRGs/Drug		
		Time	At least 6 months before claim	
		Age		
		Gender		
		Other		

© 2025 Kim DD et al. *JAMA Health Forum*.

39. Renal artery angioplasty or stenting

Recommendation	Clinical Source(s)	Low-Value Study Source(s)
Do not perform renal/visceral angioplasty or stent placement with a diagnosis of renal atherosclerosis or renovascular hypertension		Chalmers (2021)[21], Ganguli (2021)[1], Badgery-Parker (2018)[24], Schwartz (2018)[5], Carter (2017)[6], Reid (2017)[14]

Definition – Base Case		Codes	Include	Exclude
**Numerator**	Patients that received a renal/visceral angioplasty or stent placement	ICD-10	ICD-10-PCS: 027x, 037x, 047x, 057x, 067x, 4A12XSH, B20x, B21x, B30x, B31x, B40x, B41x, B508x, B509x, B518x, B519x	
		BETOS		
		CPT/HCPCS		
		MS-DRGs/Drug		
		Time	On claim	
		Age		
		Gender		
		Other		
**Denominator**	Patients with a diagnosis of renal atherosclerosis or renovascular hypertension and without fibromuscular dysplasia	ICD-10	I150, I701	I77.3
		BETOS		
		CPT/HCPCS		
		MS-DRGs/Drug		
		Time	On claim	On claim
		Age		
		Gender		
		Other		

Definition – Sensitivity Analysis		Codes	Include	Exclude
**Numerator**	Patients that received a renal/visceral angioplasty or stent placement	ICD-10	ICD-10-PCS: 027x, 037x, 047x, 057x, 067x, 4A12XSH, B20x, B21x, B30x, B31x, B40x, B41x, B508x, B509x, B518x, B519x	
		BETOS		
		CPT/HCPCS		
		MS-DRGs/Drug		

© 2025 Kim DD et al. *JAMA Health Forum*.

		Time	On claim	
		Age		
		Gender		
		Other		
**Denominator**	Patients with a diagnosis of renal atherosclerosis or renovascular hypertension and without fibromuscular dysplasia	ICD-10	I150, I701, H35.17, I10, I11, I12, I13, I15, I16, I67.4, N26.2	I77.3
		BETOS		
		CPT/HCPCS		
		MS-DRGs/Drug		
		Time	On claim	On claim
		Age		
		Gender		
		Other		

© 2025 Kim DD et al. *JAMA Health Forum*.

40. Stress test for stable coronary disease

Recommendation	Clinical Source(s)	Low-Value Study Source(s)
Do not perform stress testing not associated with inpatient or emergency care for patients with an established diagnosis of acute myocardial infraction	CW American Society of Nuclear Cardiology (2012)	Schwartz (2018)[5], Reid (2017)[14]

Definition – Base Case		Codes	Include	Exclude
**Numerator**	Patients that receive stress testing, cardiac MRI, CT angiography and was not associated with inpatient or emergency care	ICD-10		
		BETOS		
		CPT/HCPCS	75574, 78451-78454, 78460, 78461, 78464, 78465, 78472, 78473, 78481, 78483, 78491, 78492, 93015-93018, 93350, 93351	All inpatient<br>Emergency care: 99281-99285, 99288
		MS-DRGs/Drug		
		Time	On claim	On claim
		Age		
		Gender		
		Other		
**Denominator**	Patients with ischemic heart disease or acute myocardial infarction diagnosis	ICD-10	I21, I22, I24.8, I24.9, I25	
		BETOS		
		CPT/HCPCS		
		MS-DRGs/Drug		
		Time	At least 6 months before claim	
		Age		
		Gender		
		Other		

Definition – Sensitivity Analysis		Codes	Include	Exclude
**Numerator**	Patients that receive stress testing, cardiac MRI, CT angiography and was not	ICD-10		
		BETOS		
		CPT/HCPCS	75574, 78451-78454, 78460, 78461, 78464, 78465, 78472, 78473, 78481,	All inpatient<br>Emergency care: 99281-99285, 99288

© 2025 Kim DD et al. *JAMA Health Forum*.

	associated with inpatient or emergency care		78483, 78491, 78492, 93015-93018, 93350, 93351	
		MS-DRGs/Drug		
		Time	On claim	On claim
		Age		
		Gender		
		Other		
**Denominator**	Patients with ischemic heart disease or acute myocardial infarction diagnosis	ICD-10	I21, I22, I24.8, I24.9, I25	
		BETOS		
		CPT/HCPCS		
		MS-DRGs/Drug		
		Time	At least 3 months before claim	
		Age		
		Gender		
		Other		

© 2025 Kim DD et al. *JAMA Health Forum*.

41. Arthroscopic surgery of the knee

Recommendation	Clinical Source(s)	Low-Value Study Source(s)
Do not perform arthroscopic debridement / chondroplasty of the knee for patients with diagnosis of osteoarthritis or chondromalacia and no meniscal tears	CW, American Academy of Orthopaedic Surgeons (2020), CW Australian Rheumatology (2018)	Chalmers (2021)[21], Ganguli (2021)[1], Badgery-Parker (2018)[24], Schwartz (2018)[5], Reid (2017)[14], Charlesworth (2016)[15]

Definition – Base Case		Codes	Include	Exclude
**Numerator**	Patients that received arthroscopic debridement/ chondroplasty of the knee	ICD-10		
		BETOS		
		CPT/HCPCS	29877, 29879, 29880, 29881, G0289	
		MS-DRGs/Drug		
		Time	On claim	
		Age		
		Gender		
		Other		
**Denominator**	Patients with diagnosis of osteoarthritis or chondromalacia and without meniscal tears	ICD-10	M15, M17, M19.9, M22.4, M94.2	M23.2, S83.2, S83.3
		BETOS		
		CPT/HCPCS		
		MS-DRGs/Drug		
		Time	On claim	On claim
		Age		
		Gender		
		Other		

Definition – Sensitivity Analysis		Codes	Include	Exclude
**Numerator**	Patients that received arthroscopic debridement/ chondroplasty of the knee	ICD-10		
		BETOS		
		CPT/HCPCS	29877, 29879, 29880, 29881, G0289	
		MS-DRGs/Drug		
		Time	On claim	
		Age		
		Gender		

© 2025 Kim DD et al. *JAMA Health Forum*.

		Other		
**Denominator**	Patients with diagnosis of osteoarthritis or chondromalacia and without meniscal tears	ICD-10	M15, M17, M19.9, M22.4, M94.2	M23.2, S83.2, S83.3
		BETOS		
		CPT/HCPCS		
		MS-DRGs/Drug		
		Time	Within the past 1 year	On claim
		Age		
		Gender		
		Other		

© 2025 Kim DD et al. *JAMA Health Forum*.

42. Laminectomy/spinal fusion

Recommendation	Clinical Source(s)	Low-Value Study Source(s)
Do not perform spinal fusion or laminectomy		Fleming (2022)[23], Chalmers (2021)[21], Koehlmoos (2019)[18], Carter (2017)[6]

Definition – Base Case		Codes	Include	Exclude
Numerator	Patients that received laminectomy or spinal fusion	ICD-10	0RG6*, 0RG7*, 0RG8*, 0RGA*, 0SG0*, 0SG1*, 0SG3*, 0SR0*, 0SR2*, 0SR3*, 0SR4*, 0RB30ZZ, 0RB33ZZ, 0RB34ZZ, 0RB50ZZ, 0RB53ZZ, 0RB54ZZ, 0RB90ZZ, 0RB93ZZ, 0RB94ZZ, 0RBB0ZZ, 0RBB3ZZ, 0RBB4ZZ, 0RT30ZZ, 0RT40ZZ, 0RT50ZZ, 0RT90ZZ, 0RTB0ZZ, 0SB20ZZ, 0SB23ZZ, 0SB24ZZ, 0SB40ZZ, 0SB43ZZ, 0SB44ZZ, 0SG8*, 0ST20ZZ, 0ST40ZZ	
		BETOS		
		CPT/HCPCS	22533, 22534, 22558, 22630, 0275T, 63005, 63012, 63017, 63030, 63035, 63042, 63047, 63200, 63267, 63272, 63173, 63185, 63190, 63191, 22612, 22633	
		MS-DRGs/Drug	453, 454, 455, 459, 460	
		Time	On claim	
		Age		
		Gender		
		Other		
Denominator	Patients without at least two diagnoses indicating sciatica or radicular pain in the past 30 days and without herniated disc, spondylolisthesis, pain in the foot, or congenital malformations of the spine within the past 12 months	ICD-10		**Sciatica/Radicular pain:** M541x, M543x **Herniated disc:** M4640, M4645, M4647, M5000, M5080, M5090, M5104, M5105, M5106, M5124, M5125, M5126, M5127, M5134, M5135, M5136, M5137, M5144, M5145, M5146, M5147, M5184, M5185, M5186, M5187, M519, M961 **Spondylisthesis:** M431

© 2025 Kim DD et al. *JAMA Health Forum*.

				**Pain in the foot:** M796<br>**Congenital malformations of the spine:** Q76
		BETOS		
		CPT/HCPCS		
		MS-DRGs/Drug		
		Time		**Sciatica/Radicular pain:** At least two diagnoses within the past 30 days on two different claims<br>**All others:** within the past 12 months
		Age		
		Gender		
		Other		

**Definition – Sensitivity Analysis**		**Codes**	**Include**	**Exclude**
**Numerator**	Patients that received laminectomy or spinal fusion	ICD-10	0RG6*, 0RG7*, 0RG8*, 0RGA*, 0SG0*, 0SG1*, 0SG3*, 0SR0*, 0SR2*, 0SR3*, 0SR4*, 0RB30ZZ, 0RB33ZZ, 0RB34ZZ, 0RB50ZZ, 0RB53ZZ, 0RB54ZZ, 0RB90ZZ, 0RB93ZZ, 0RB94ZZ, 0RBB0ZZ, 0RBB3ZZ, 0RBB4ZZ, 0RT30ZZ, 0RT40ZZ, 0RT50ZZ, 0RT90ZZ, 0RTB0ZZ, 0SB20ZZ, 0SB23ZZ, 0SB24ZZ, 0SB40ZZ, 0SB43ZZ, 0SB44ZZ, 0SG8*, 0ST20ZZ, 0ST40ZZ	
		BETOS		
		CPT/HCPCS	22533, 22534, 22558, 22630, 0275T, 63005, 63012, 63017, 63030, 63035, 63042, 63047, 63200, 63267, 63272, 63173, 63185, 63190, 63191, 22612, 22633	
		MS-DRGs/Drug	453, 454, 455, 459, 460	
		Time	On claim	
		Age		
		Gender		
		Other		
**Denominator**		ICD-10		M541x, M543x

© 2025 Kim DD et al. *JAMA Health Forum*.

	Patients without at least two diagnoses indicating sciatica or radicular pain in the past 30 days	BETOS		
		CPT/HCPCS		
		MS-DRGs/Drug		
		Time		At least two diagnoses within the past 30 days on two different claims
		Age		
		Gender		
		Other		

© 2025 Kim DD et al. *JAMA Health Forum*.

43. Spinal injection for low back pain (LBP)

Recommendation	Clinical Source(s)	Low-Value Study Source(s)
Do not perform outpatient epidural, facet, or trigger point injections for lower back pain, excluding etanercept, for patients with no radiculopathy diagnoses		Ganguli (2021)[1], Sanghavi (2021)[3], Schwartz (2018)[5], Reid (2017)[14], Charlesworth (2016)[15]

Definition – Base Case		Codes	Include	Exclude
**Numerator**	Patients that received epidural, facet, or trigger point injections not in an inpatient setting and excluding etanercept injection	ICD-10		
		BETOS		
		CPT/HCPCS	62311, 64483, 20552, 20553, 64493, 64475	All inpatient<br>J1438
		MS-DRGs/Drug		
		Time	On claim	On claim
		Age		
		Gender		
		Other		
**Denominator**	Patients with lower back pain without radiculopathy	ICD-10	M43.0, M43.1, M43.27, M43.28, M46.46, M46.47, M47.20, M47.26, M47.27, M47.28, M47.816, M47.817, M47.818, M47.819, M47.896, M47.897, M47.898, M47.899, M47.9, M48.00, M48.06, M48.07, M51.16, M51.17, M51.26, M51.27, M51.3, M51.86, M51.87, M51.9, M53.2X7, M53.2X8, M53.3, M53.86, M53.87, M53.88, M54.5, M54.89, M54.9, M96.1, M99.03, M99.04, M99.23, M99.33, M99.43, M99.53, M99.63, M99.73, M99.83, M99.84, Q76.2, S33.5, S33.6, S33.8, S33.9	M47.16, M51.06, M51.9, M54.14, M54.15, M54.16, M54.17, M54.3, M54.4
		BETOS		
		CPT/HCPCS		
		MS-DRGs/Drug		
		Time	On claim	On claim

© 2025 Kim DD et al. *JAMA Health Forum*.

		Age		
		Gender		
		Other		

**Definition – Sensitivity Analysis**		**Codes**	**Include**	**Exclude**
**Numerator**	Patients that received epidural, facet, or trigger point injections not in an inpatient setting and excluding etanercept injection	ICD-10		
		BETOS		
		CPT/HCPCS	62311, 64483, 20552, 20553, 64493, 64475	All inpatient J1438
		MS-DRGs/Drug		
		Time	On claim	On claim
		Age		
		Gender		
		Other		
**Denominator**	Patients with lower back pain	ICD-10	M43.0, M43.1, M43.27, M43.28, M46.46, M46.47, M47.20, M47.26, M47.27, M47.28, M47.816, M47.817, M47.818, M47.819, M47.896, M47.897, M47.898, M47.899, M47.9, M48.00, M48.06, M48.07, M51.16, M51.17, M51.26, M51.27, M51.3, M51.86, M51.87, M51.9, M53.2X7, M53.2X8, M53.3, M53.86, M53.87, M53.88, M54.5, M54.89, M54.9, M96.1, M99.03, M99.04, M99.23, M99.33, M99.43, M99.53, M99.63, M99.73, M99.83, M99.84, Q76.2, S33.5, S33.6, S33.8, S33.9	
		BETOS		
		CPT/HCPCS		
		MS-DRGs/Drug		
		Time	Within the past 14 days	
		Age		
		Gender		
		Other		

© 2025 Kim DD et al. *JAMA Health Forum*.

44. Vertebroplasty/kyphoplasty

Recommendation	Clinical Source(s)	Low-Value Study Source(s)
Do not perform vertebroplasty or kyphoplasty for osteoporotic vertebral fracture		Chalmers (2021)[21], Ganguli (2021)[1], Badgery-Parker (2018)[24], Schwartz (2018)[5], Carter (2017)[6], Reid (2017)[14]

Definition – Base Case		Codes	Include	Exclude
**Numerator**	Patients that received vertebroplasty or kyphoplasty	ICD-10	ICD-10-PCS: 01U13JZ, 01U14JZ, 0PU33JZ, 0PU34JZ, 0RU13JZ, 0RU14JZ, 0RU33JZ, 0RU34JZ, 0RU63JZ, 0RU64JZ, 0RU93JZ, 0RU94JZ, XNU4356, 01UB3JZ, 01UB4JZ, 0QU03JZ, 0QU04JZ, 0SU03JZ, 0SU04JZ, 0SU23JZ, 0SU24JZ, XNU0356, 0QU13JZ, 0QU14JZ	
		BETOS		
		CPT/HCPCS		
		MS-DRGs/Drug		
		Time	On claim	
		Age		
		Gender		
		Other		
**Denominator**	Patients with osteoporosis and with vertebral fractures and without bone cancer, myeloma, hemangioma	ICD-10	**Vertebral fracture:** M48.5, M80.08, M80.88, M84.48, M84.58, M84.68, S22.0, S32.0<br>**Osteoporosis:** M81	C41.2, C79.5, C7B.03, C90.0, D16.6, D18.09, D47.Z9, D48.0, D49.2
		BETOS		
		CPT/HCPCS		
		MS-DRGs/Drug		
		Time	**Vertebral fracture:** On claim<br>**Osteoporosis:** Within the past 1 year	Within the past 1 year
		Age		
		Gender		
		Other		

© 2025 Kim DD et al. *JAMA Health Forum*.

Definition – Sensitivity Analysis		Codes	Include	Exclude
**Numerator**	Patients that received vertebroplasty or kyphoplasty	ICD-10	ICD-10-PCS: 01U13JZ, 01U14JZ, 0PU33JZ, 0PU34JZ, 0RU13JZ, 0RU14JZ, 0RU33JZ, 0RU34JZ, 0RU63JZ, 0RU64JZ, 0RU93JZ, 0RU94JZ, XNU4356, 01UB3JZ, 01UB4JZ, 0QU03JZ, 0QU04JZ, 0SU03JZ, 0SU04JZ, 0SU23JZ, 0SU24JZ, XNU0356, 0QU13JZ, 0QU14JZ	
		BETOS		
		CPT/HCPCS		
		MS-DRGs/Drug		
		Time	On claim	
		Age		
		Gender		
		Other		
**Denominator**	Patients with osteoporosis and with vertebral fractures	ICD-10	**Vertebral fracture:** M48.5, M80.08, M80.88, M84.48, M84.58, M84.68, S22.0, S32.0 **Osteoporosis:** M81	
		BETOS		
		CPT/HCPCS		
		MS-DRGs/Drug		
		Time	**Vertebral fracture:** On claim **Osteoporosis:** Within the past 1 year	
		Age		
		Gender		
		Other		

© 2025 Kim DD et al. *JAMA Health Forum*.

45. Feeding tubes in patients with dementia

Recommendation	Clinical Source(s)	Low-Value Study Source(s)
Do not use percutaneous feeding tubes in patients with advanced dementia	CW American Academy of Hospice and Palliative Medicine (2021/2013), CW American Geriatrics Society (2013)	Ganguli (2021)[1], Schpero (2017)[9], Colla (2015)[10]

Definition – Base Case		Codes	Include	Exclude
**Numerator**	Patients that received a feeding tube	ICD-10	ICD-10-PCS: 0D16074 0D160J4, 0D160K4, 0D160Z4, 0D163J4, 0D16474, 0D164J4, 0D164K4, 0D164Z4, 0D16874, 0D168J4, 0D168K4, 0D168Z4, 0DH60UZ, 0DH63UZ, 0DH64UZ, 0DW04UZ, 0DW08UZ	
		BETOS		
		CPT/HCPCS	43246, 43653, 43750, 43830, 43832, 44372, 44373, 74350, 49440, 49411, 49446, 49450, 49451, 49452, 43760, 43761	
		MS-DRGs/Drug		
		Time	On claim	
		Age		
		Gender		
		Other		
**Denominator**	Patients with at least two dementia diagnoses	ICD-10	F01.50, F01.51, F02.80, F02.81, F03.90, F05, F10.27, G13.2, G13.8, G30, G31.01, G31.09, G31.1, G31.2, G31.83, G91.4, G94	
		BETOS		
		CPT/HCPCS		
		MS-DRGs/Drug		
		Time	At least two diagnoses in all past data	
		Age		
		Gender		
		Other		

© 2025 Kim DD et al. *JAMA Health Forum*.

Definition – Sensitivity Analysis		Codes	Include	Exclude
**Numerator**	Patients that received a feeding tube	ICD-10	ICD-10-PCS: 0D16074 0D160J4, 0D160K4, 0D160Z4, 0D163J4, 0D16474, 0D164J4, 0D164K4, 0D164Z4, 0D16874, 0D168J4, 0D168K4, 0D168Z4, 0DH60UZ, 0DH63UZ, 0DH64UZ, 0DW04UZ, 0DW08UZ	
		BETOS		
		CPT/HCPCS	43246, 43653, 43750, 43830, 43832, 44372, 44373, 74350, 49440, 49411, 49446, 49450, 49451, 49452, 43760, 43761	
		MS-DRGs/Drug		
		Time	On claim	
		Age		
		Gender		
		Other		
**Denominator**	Patients with dementia diagnosis	ICD-10	F01.50, F01.51, F02.80, F02.81, F03.90, F05, F10.27, G13.2, G13.8, G30, G31.01, G31.09, G31.1, G31.2, G31.83, G91.4, G94	
		BETOS		
		CPT/HCPCS		
		MS-DRGs/Drug		
		Time	All past data	
		Age		
		Gender		
		Other		

© 2025 Kim DD et al. *JAMA Health Forum*.

46. Carotid endarterectomy (CEA)

Recommendation	Clinical Source(s)	Low-Value Study Source(s)
Do not perform carotid endarterectomy (CEA), not associated with an ER visit, for patients without a history of stroke or TIA and without stroke, TIA, or focal neurological symptoms	CW Canadian Society for Vascular Surgery (2021), CW Australian and New Zealand Association of Neurologists (2016), CW American Academy of Neurology (2013)	Chalmers (2021)[21], Badgery-Parker (2018)[24], Schwartz (2018)[5]

Definition – Base Case		Codes	Include	Exclude
**Numerator**	Patients that received carotid endarterectomy and was not associated with emergency care	ICD-10		
		BETOS		
		CPT/HCPCS	35301	Emergency care: 99281-99285, 99288
		MS-DRGs/Drug		
		Time	On claim	On claim
		Age		
		Gender		
		Other		
**Denominator**	Patients without a history of stroke or TIA, stroke or TIA, or focal neurological symptoms	ICD-10		G45, G46.0, G46.1, G46.2, G97.3, H34, H35.82, I60, I61, I63, I66, I67.84, I67.89, I97.81, I97.82, R20, R25, R26, R27, R29, R41.4, R43, R47, R68.3, Z86.73
		BETOS		
		CPT/HCPCS		
		MS-DRGs/Drug		
		Time		All past data
		Age		
		Gender		
		Other		

No definition for sensitivity analysis

© 2025 Kim DD et al. *JAMA Health Forum*.

47. Hysterectomy

Recommendation	Clinical Source(s)	Low-Value Study Source(s)
Do not perform hysterectomy for benign disease		Chalmers (2021)[21], Koehlmoos (2019)[18], Carter (2017)[6]

Definition – Base Case		Codes	Include	Exclude
**Numerator**	Patients that received hysterectomy	ICD-10	0UT9x, 0UT4x	
		BETOS		
		CPT/HCPCS	58150, 58152, 58180, 58200, 52810, 58260, 58262, 58263, 58267, 58270, 58275, 58280, 58285, 58290, 58291, 58292, 59293, 59294, 58541, 58542, 58543, 58544, 58548, 58550, 58552, 58553, 58554, 58570, 58571, 58572, 58573	
		MS-DRGs/Drug		
		Time		
		Age		
		Gender		
		Other		
**Denominator**	Patients that are female and age 50 or older and without a malignancy or carcinoma in situ	ICD-10		C51x - C58x, C7982, D06x - D07x
		BETOS		
		CPT/HCPCS		
		MS-DRGs/Drug		734 – 756
		Time		On claim
		Age	50+	
		Gender	Female	
		Other		

Definition – Sensitivity Analysis		Codes	Include	Exclude
**Numerator**	Patients that received hysterectomy	ICD-10		
		BETOS		

© 2025 Kim DD et al. *JAMA Health Forum*.

		CPT/HCPCS	58150, 58152, 58180, 58200, 52810, 58260, 58262, 58263, 58267, 58270, 58275, 58280, 58285, 58290, 58291, 58292, 59293, 59294, 58541, 58542, 58543, 58544, 58548, 58550, 58552, 58553, 58554, 58570, 58571, 58572, 58573	
		MS-DRGs/Drug		
		Time		
		Age		
		Gender		
		Other		
**Denominator**	Patients that are female and age 50 or older and without a malignancy	ICD-10		C51x - C58x, C7982
		BETOS		
		CPT/HCPCS		
		MS-DRGs/Drug		734 – 756
		Time		On claim
		Age	50+	
		Gender	Female	
		Other		

© 2025 Kim DD et al. *JAMA Health Forum*.

eReferences

1. Ganguli I, Morden NE, Yang CW, Crawford M, Colla CH. Low-Value Care at the Actionable Level of Individual Health Systems. JAMA Intern Med 2021;181(11):1490-1500. (In eng). DOI: 10.1001/jamainternmed.2021.5531.
2. Park S, Jung J, Burke RE, Larson EB. Trends in Use of Low-Value Care in Traditional Fee-for-Service Medicare and Medicare Advantage. JAMA Netw Open 2021;4(3):e211762. (In eng). DOI: 10.1001/jamanetworkopen.2021.1762.
3. Sanghavi P, McWilliams JM, Schwartz AL, Zaslavsky AM. Association of Low-Value Care Exposure With Health Care Experience Ratings Among Patient Panels. JAMA Intern Med 2021;181(7):941-948. (In eng). DOI: 10.1001/jamainternmed.2021.1974.
4. Bouck Z, Ferguson J, Ivers NM, et al. Physician Characteristics Associated With Ordering 4 Low-Value Screening Tests in Primary Care. JAMA Netw Open 2018;1(6):e183506. (In eng). DOI: 10.1001/jamanetworkopen.2018.3506.
5. Schwartz AL, Zaslavsky AM, Landon BE, Chernew ME, McWilliams JM. Low-Value Service Use in Provider Organizations. Health Serv Res 2018;53(1):87-119. (In eng). DOI: 10.1111/1475-6773.12597.
6. Carter EA, Morin PE, Lind KD. Costs and Trends in Utilization of Low-value Services Among Older Adults With Commercial Insurance or Medicare Advantage. Med Care 2017;55(11):931-939. (In eng). DOI: 10.1097/mlr.0000000000000809.
7. McAlister FA, Lin M, Bakal J, Dean S. Frequency of low-value care in Alberta, Canada: a retrospective cohort study. BMJ Qual Saf 2018;27(5):340-346. (In eng). DOI: 10.1136/bmjqs-2017-006778.
8. Pendrith C, Bhatia M, Ivers NM, et al. Frequency of and variation in low-value care in primary care: a retrospective cohort study. CMAJ Open 2017;5(1):E45-e51. (In eng). DOI: 10.9778/cmajo.20160095.
9. Schpero WL, Morden NE, Sequist TD, Rosenthal MB, Gottlieb DJ, Colla CH. For Selected Services, Blacks And Hispanics More Likely To Receive Low-Value Care Than Whites. Health Aff (Millwood) 2017;36(6):1065-1069. (In eng). DOI: 10.1377/hlthaff.2016.1416.
10. Colla CH, Morden NE, Sequist TD, Schpero WL, Rosenthal MB. Choosing wisely: prevalence and correlates of low-value health care services in the United States. J Gen Intern Med 2015;30(2):221-8. (In eng). DOI: 10.1007/s11606-014-3070-z.
11. Kool RB, Verkerk EW, Meijs J, et al. Assessing volume and variation of low-value care practices in the Netherlands. Eur J Public Health 2020;30(2):236-240. (In eng). DOI: 10.1093/eurpub/ckz245.
12. Rhyan C, Beaudin-Seiler B, Fendrick M, Miller G, Budros M. The "Top 5" Low- and High-Value Services: Trends in Health Care Spending Among the Privately Insured, 2014-2016. Research Consortium for Health Care Value Assessment. (https://www.hcvalueassessment.org/publications).
13. Shahzad M, Song Z, Chernew ME, Fendrick AM. Changes in use of low-value services during the COVID-19 pandemic. Am J Manag Care 2022;28(11):600-604. (In eng). DOI: 10.37765/ajmc.2022.89031.
14. Reid RO, Rabideau B, Sood N. Impact of consumer-directed health plans on low-value healthcare. Am J Manag Care 2017;23(12):741-748. (In eng).
15. Charlesworth CJ, Meath TH, Schwartz AL, McConnell KJ. Comparison of Low-Value Care in Medicaid vs Commercially Insured Populations. JAMA Intern Med 2016;176(7):998-1004. (In eng). DOI: 10.1001/jamainternmed.2016.2086.

© 2025 Kim DD et al. *JAMA Health Forum*.

16. Rosenthal MB, Colla CH, Morden NE, et al. Overuse and insurance plan type in a privately insured population. Am J Manag Care 2018;24(3):140-146. (In eng).
17. Mafi JN, Russell K, Bortz BA, Dachary M, Hazel WA, Jr., Fendrick AM. Low-Cost, High-Volume Health Services Contribute The Most To Unnecessary Health Spending. Health Aff (Millwood) 2017;36(10):1701-1704. (In eng). DOI: 10.1377/hlthaff.2017.0385.
18. Koehlmoos TP, Madsen CK, Banaag A, Haider AH, Schoenfeld AJ, Weissman JS. Assessing Low-Value Health Care Services In The Military Health System. Health Aff (Millwood) 2019;38(8):1351-1357. (In eng). DOI: 10.1377/hlthaff.2019.00252.
19. Oakes AH, Chang HY, Segal JB. Systemic overuse of health care in a commercially insured US population, 2010-2015. BMC Health Serv Res 2019;19(1):280. (In eng). DOI: 10.1186/s12913-019-4079-0.
20. Segal JB, Bridges JF, Chang HY, et al. Identifying possible indicators of systematic overuse of health care procedures with claims data. Med Care 2014;52(2):157-63. (In eng). DOI: 10.1097/mlr.0000000000000052.
21. Chalmers K, Smith P, Garber J, et al. Assessment of Overuse of Medical Tests and Treatments at US Hospitals Using Medicare Claims. JAMA Netw Open 2021;4(4):e218075. (In eng). DOI: 10.1001/jamanetworkopen.2021.8075.
22. Barnett ML, Linder JA, Clark CR, Sommers BD. Low-Value Medical Services in the Safety-Net Population. JAMA Intern Med 2017;177(6):829-837. (In eng). DOI: 10.1001/jamainternmed.2017.0401.
23. Fleming C, Shin E, Powell R, et al. Updating a Claims-Based Measure of Low-Value Services Applicable to Medicare Fee-for-Service Beneficiaries. J Gen Intern Med 2022;37(13):3453-3461. (In eng). DOI: 10.1007/s11606-022-07654-7.
24. Badgery-Parker T, Pearson SA, Chalmers K, et al. Low-value care in Australian public hospitals: prevalence and trends over time. BMJ Qual Saf 2019;28(3):205-214. (In eng). DOI: 10.1136/bmjqs-2018-008338.

© 2025 Kim DD et al. *JAMA Health Forum*.
