# Supplementary material for: Projected Savings From Reducing Low-Value Services in Medicare
Source: JAMA Health Forum. 2025 Aug 1;6(8):e253050. doi: 10.1001/jamahealthforum.2025.3050 (PMC12317346; doi:10.1001/jamahealthforum.2025.3050)
Supplement: Supplement 2. — Data Sharing Statement [file jamahealthforum-e253050-s002.pdf]

Data Sharing Statement

Kim. Projected Savings From Reducing Low-Value Services in Medicare. *JAMA Health Forum*. Published August 01, 2025. doi:10.1001/jamahealthforum.2025.3050

Data
Data available: No

Additional Information
Explanation for why data not available: This study used a Limited Data Set (LDS) from the Centers for Medicare & Medicaid Services (CMS). Access to the data is subject to a Data Use Agreement with CMS and cannot be shared publicly. Researchers may request access to the data directly from CMS.
